# Genome-Wide Association and Pathway Analysis of Carcass and Meat Quality Traits in Karachai Young Goats

**DOI:** 10.3390/ani13203237

**Published:** 2023-10-17

**Authors:** Marina Selionova, Magomet Aibazov, Alexander Sermyagin, Anna Belous, Tatiana Deniskova, Tatiana Mamontova, Ekaterina Zharkova, Natalia Zinovieva

**Affiliations:** 1 Subdepartment of Animal Breeding, Genetics and Biotechnology, Moscow Timiryazev Agricultural Academy, Russian State Agrarian University, Timiryazevskaya Street, 41, 127343 Moscow, Russia; m_selin@mail.ru (M.S.); info@fnac.center (T.M.); 2 North Caucasian Agrarian Center, Zootechnicheski 15, 355017 Stavropol, Russia; velikii-1@yandex.ru; 3 L. K. Ernst Federal Research Center for Animal Husbandry, Dubrovitsy 60, 142132 Moscow, Russia; alex_sermyagin85@mail.ru (A.S.); abelous.vij@ya.ru (A.B.); horarka@yandex.ru (T.D.); n_zinovieva@mail.ru (N.Z.)

**Keywords:** GWAS, SNP, QTL, candidate genes, meat, carcass, Karachai goats

## Abstract

**Simple Summary:**

Goats with diverse economic phenotypic traits play an important role in animal husbandry. However, the genetic mechanisms underlying complex phenotypic traits are unclear in goats. Genomic studies of variations provided a lens to identify functional genes. The work aimed to search for candidate genes related to body measurements and body weight of Karachai goats based on genome-wide associations. Structural annotation of the identified genomic regions showed that they regulate the processes of transcription, cell proliferation, angiogenesis, body growth, fatty acid and lipid metabolism, nervous system development, and spermatogenesis. For further use in breeding, SNPs were selected that were significant for live weight at four and eight months. It was located in the vicinity of structural genes. An experimental PCR-RV test system was developed and tested for the genotyping of significant SNPs. Our study found a set of new DNA markers for genetic improvement in meat goats and provided novel insights into the genetic mechanisms of complex traits.

**Abstract:**

Goats with diverse economic phenotypic traits play an important role in animal husbandry. However, the genetic mechanisms underlying complex phenotypic traits are unclear in goats. Genomic studies of variations provided a lens to identify functional genes. The work aimed to search for candidate genes related to body measurements and body weight of Karachai goats and develop an experimental PCR-RV test system for genotyping significant SNPs. Comparison of GWAS results for ages 4 and 8 months revealed 58 common SNPs for significant genotypes. 11 common SNPs were identified for body weight, 4 SNPs—for group of traits withers height, rump height, body length, 2 SNPs—for withers height and rump height, 1 SNP—for body length and chest depth. Structural annotation of genomic regions covering a window of ±0.20 Mb showed the presence of 288 genes; 52 of them had the described functions in accordance with gene ontology. The main molecular functions of proteins encoded by these genes are the regulation of transcription, cell proliferation, angiogenesis, body growth, fatty acid and lipid metabolism, nervous system development, and spermatogenesis. SNPs common to body weight and localized within a window of ±200 kb from the structural genes *CRADD*, *HMGA2*, *MSRB3*, *FUT8*, *MAX*, and *RAB15* were selected to create a test system. The study of meat productivity after slaughter and chemical analysis of muscle tissue in Karachai goats at the age of 8 months of different genotypes according to the identified SNPs revealed that rs268269710 is the most promising for further research and use in breeding. The GG genotype is associated with a larger live weight of animals, a larger carcass yield, the content of the boneless part in it, and the ratio of protein and adipose tissue in meat preferred for dietary nutrition. These results will contribute to the genetic improvement of Karachai goats.

## 1. Introduction

Goats are known as one of the most common farm animals. Their high adaptive abilities to various, and sometimes extreme, living conditions have formed many breeds of different productivity trends in more than 195 countries around the world [1]. The global number of goats is about 1.2 billion and has a positive tendency to increase [2,3]. The interest in goat breeding is due not only to the variety of products received from them—milk, wool, meat but also the ability to produce all this in ecological and geographical regions where agriculture is limited by factors such as insufficient moisture, high temperature, difficult terrain [4,5]. This explains that out of 635 goat breeds, only 38 breeds are classified as cross-border. The rest are indigenous and adapted to difficult breeding conditions (http://www.fao.org/dad-is, accessed on 9 June 2022). Local breeds are most widespread in Central, South and East Asia and Africa and are a valuable source of animal products for developing countries [6,7].

Long-term breeding to improve productivity while maintaining high adaptive qualities of goats affected changes in their genome, as evidenced by several studies of modern and ancient goat DNA [7,8]. The successful implementation of several international projects on goat genome research, the development of Goat 50K BeadChip and the use of genome-wide associative analysis (GWAS) provided significant progress in the search for breeding prints and the identification of SNPs associated with different indicators of goat productivity [9,10,11]. The following groups of genes have been identified: controlling signs of lactic acid [12,13,14,15,16], down productivity and wool color [17,18], reproduction [19,20,21,22], as well as adaptations [23,24].

To increase meat productivity and include valuable genotypes in breeding programs for further replication, a search for genomic associations was performed, and candidate genes for body parameters and live weight of goats were established. Thus, in goats of the dazu black goat breed, genotyped using Goat 50K BeadChip and sequencing of the complete genome using GWAS, it was revealed that the genes *PRDM6*, *PSTPIP2*, *SUN3*, *MANEA*, *CDKAL1*, *CDH9*, *CBLN4*, *SOX4*, *SGCG*, *FIG9*, *CNTFR*, *RPP25L*, *FGF9*, *SIPA1L* and *CCL19*, are associated with the height and length of the body, with the width and depth of the chest, the width of the hips and the live weight of the young. Functional annotation of these genes has shown that they regulate the immune response, cell division processes, and skeletal muscle development [25,26].

In Kashmiri (downy) goats, a connection was found with the live weight and height, body length, depth, and chest width of the genes *KDM6A*, *AKAP12* and *SNX29*, which regulate the processes of proliferation of muscle tissue cells [27,28,29].

The association of the genes *DKK2*, *TBCK*, *FGF*, and *ANK2*, which are involved in the processes of embryonic development and regulation of cell proliferation and growth, has been established with the body size in goats of seven breeds bred in Pakistan [30].

Candidate genes associated with body type in Mursiana granadina goats have been identified, including genes associated with collagen synthesis (*ATF3*, *ADAMTS14* and *COL14A1*), general growth and development (*CGGBP1WNT5A*, *DNAH14*), limb development (*ECEL1* and *PIEZO2*), homeostasis and bone remodeling (*PTH1R*, *CDH11*, *SPATA4* and *EPHA3*) [31].

Three SNPs associated with body weight were identified in Beetal goats, which were located within the *BTAF1*, *NTM* and *GRID1* genes. In addition, some of the established SNPs were localized near the genes *CEP78*, *ROBO1*, *ZFP36L2*, *SPTLC3*, *CTR 9* and *ZFHX3*. Functional annotation of these genes has shown that they are involved either in growth, development or in both basic cell functions from embryonic to adult life of an animal [32].

Several studies performed on different breeds of goats have established a connection with the live weight and growth dynamics of such genes as *PRLR*, *MYLK* and *CADM2* [33,34,35], *IGF-I* [36], which are more associated with endocrine and autocrine effects, enzymatic reactions in muscle tissue, and also with synaptic interactions in cells.

In Russia, there has recently been a growing interest in breeding dairy and meat-dairy goats. Goats of double productivity are most widespread in Altai, Tyva, Khakassia and the North Caucasus [37]. One of the breeds of interest for the production of milk and meat is the Karachai goat breed. It is bred on mountain and foothill pastures of the Caucasus, which are characterized by rich forage resources, but due to the complexity of the terrain, they are not available for other types of domestic animals. The ecological well-being of the Karachai goat breeding region makes it in demand to obtain goat meat from them, which determines the direction of breeding to improve their meat productivity [38].

Taking into account the relevance of genome-wide associative analysis, the work aimed to search for candidate genes associated with body measurements and body weight of Karachai goats to develop an experimental PCR-RT test system for genotyping significant SNPs based on functional annotation of genes, as well as to study meat productivity indicators of different genotypes.

## 2. Materials and Methods

### 2.1. Sample Collection and Phenotypic Measurements

**Figure 1:**
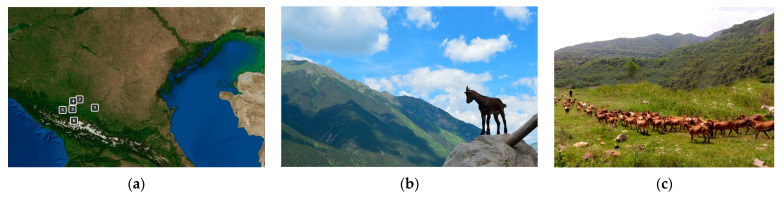
Karachai goats: (**a**) herds location 1—Piatigorsky, 2—Maysky, 3—Kyzyl Kala, 4—Darick, 5—Storozhevaya; 6—Uchkulan; (**b**) yang goat; (**c**) herd on grazing.

To obtain a representative sample of young Karachai goats and exclude kinship, 287 clinically healthy animals (74 male and 213 female goats) at four months were randomly selected in genetically isolated herds using unrelated producers and belonging to six different farmers. The location of the farm herds was chosen in such a way as to cover the entire region of breeding Karachai goats in the North Caucasus. Farms “Pyatigorsky” and “Maysky” were located at an altitude of 400–600 m above sea level, “Kyzyl-Kala”, “Storozhevaya”, “Darik”—900–1200 m, “Uchkulan”—1500 m. The distance between the farms did not exceed 350 km (Figure 1). The system of maintenance—pasture in the spring, summer, and autumn seasons, in winter—is stable also with grazing on pasture in the absence of snow cover. Depending on age, the feeding ration of young animals includes 5 to 10 kg of green mass of pasture grass of the subalpine belt, consisting of 50% of mothley grass, 40% of cereals and 10% of legumes. In winter, in addition to pasture feed, the animals received 0.8–1.2 kg of hay from cereals and legumes of subalpine meadows and 0.1–0.2 kg of compound feed concentrate.

In the selected animals at 4 and 8 months, body measurements such as rump height (RH), body length (BL), chest perimeter (CP), chest width (CW), chest depth (CD) and rump width (RW) were recorded using measuring sticks and tape, in addition, withers height (WH) were determined by weighing on an electronic scale. The data obtained are summarized in Table S1. At four months, samples of ear tissue or blood were taken into sterile tubes with a preservative for further DNA isolation.

### 2.2. Statistical Analysis

The fixed effect of each trait in the model was identified by the General Linear Model (GLM) procedure using the Statistical 10 program.

To describe the data, a mixed linear model was used, the equation of which is presented as follows:

y = HYi + Sexk + b_1_Age + animalj + e,

where: y— the phenotypic value of the i th individual the corresponding GLM (general linear model); HYi—fixed effect “herd-year” of animal birth (i = 1–10); Sexk—the fixed effect of the sex of the animal (k—male, female); Age—the regression effect of age in days at the time the i th individual was assessed; b_1_—the regression coefficient of the model; animalj—the fixed effect of i th individual (j = 1–269) weighed by covariance structure for genomic relationship matrix (GRM, N(0, Gσg 2)) built from the genomic information using VanRaden’s method [39]; e—residual effect of the model.

### 2.3. Genotyping and Quality Control of Data

Genomic DNA was isolated from ear tissue and blood samples according to the protocol of the DNA-Extran kit (Syntol, Moscow, Russia). The concentration was determined on a Qubit device (Thermofisher, Waltham, MA, USA), and samples were taken with a ratio of A260/280 in the range from 1.7 to 2.0. Genotyping was performed using GoatSNP53K Beadchip (Illumina Inc., San Diego, CA, USA). Processing of genotype data quality control was carried out using Plink 1.9 (http://zzz.bwh.harvard.edu/plink/, accessed: 9 June 2022) [40] by the following filters: (1) the frequency of calls for all SNPs for a separate sampling frame is not less than 90%; (2) the frequency of occurrence of each of the studied SNPs for all genotyped samples is not less than 90%: (3) the frequency of the minor allele for each of the studied SNPs is not less than 5%; (4) the deviation of the SNP genotypes from the Hardy-Weinberg distribution in the set test samples with a *p*-value < 10^−6^. The received data were processed by the GenomeStudio 2.0 software and converted into the Plink (.bed, .bim, .fam). The format of standard A/B genotypes has been converted to a nucleotide format corresponding to allelic variants. The data that did not pass the quality filter was not applied in further analysis. As a result, genotypes of 269 animals were used on 47,647 polymorphic sites.

To stratify data from six herds, a Principal Component Analysis (PCA) was performed using the PLINK v1.9 program.

### 2.4. Genome-Wide Association Studies

The associative search was performed using Plink, a set of tools for analyzing associations of the entire genome, namely the calculation of linear regression dependence and determination coefficients. The significance of the regression coefficients of polymorphic sites was evaluated using the Bonferroni null hypothesis test. Sites whose *p*-value was higher than the quotient of dividing the significance level by the number of polymorphic sites whose *p*-value of the test was less than 0.05 were considered potentially significant (threshold *p* < 1.05 × 10^−6^; 0.05/47647 SNP). The quantile-quantile (Q-Q) graphs were constructed by distributing the received and expected log10 (*p*-value) with coefficients λ. The distribution of associations and significant SNPs were visualized on a Manhattan graph constructed using R v. 3.5.2 R [41].

The quantile–quantile (Q–Q) plots were visualized by plotting the distribution of obtained vs. expected log10 (*p* value) with inflation factors (λ). The association map and the significant SNPs were visualized in the Manhattan plot with a threshold line. The Manhattan and Q–Q graphics were generated with R v. 3.5.2.

### 2.5. Gene Analysis

The resulting polymorphic sites were annotated with rs identifiers in accordance with the AdaptMap genome assembly and were transformed into the ARS1.2 genome assembly using the Ensembl Genes release 103 database [42].

Functional annotation and analysis of the enrichment of gene ontology (GO) terms for structural candidate genes identified in a window of ±0.2 Mb from statistically significant SNPs were used by DAVID v6.8 [43]. Significant clusters were selected based on an enrichment index of more than 1 and a *p*-value of <0.05. The Benjamin-Hochberg test was used to control the level of false deviations, defined as the expected ratio of false deviations to the total number of deviations [39].

### 2.6. Development of a Multiplex Test System

The method of hybridization-fluorescence detection in “real-time” mode based on Taq-Man technology was chosen to construct a multiplex test system [44]. Two allele-specific dyes embedded in the probe sequence were used for each locus.

The polymerase chain reaction was carried out in a final volume of 20 µL: 19 µL of a reaction mixture consisting of 20 mM (NH_4_)_2_SO_4_; 75 mM Tris-HCl; pH = 8.8; 0.1% (*v*/*v*) Tween 20; 2.5 mM MgCl_2_; 0.25 mM dNTP, 0.15 units Taq-DNA polymerase, and 1 µL (~10–200 ng) of the genomic DNA under study. After initial denaturation (95 °C, 7 min), 40 amplification cycles were performed (95 °C, 25 s.; 58 °C, 35 s.; 72 °C, 4 s).

Validation of test systems was performed by comparing the results of genotyping on 46 goats obtained using Goat 60K BeadChip and developed test systems. The results of genotyping of all 46 animals obtained using the two above-mentioned methodological approaches completely coincided.

### 2.7. Meat Productivity and Meat Quality Analysis

Three animals of each genotype were selected from 35 goats aged eight months genotyped using the developed test system. The selected goats were examined for signs of meat productivity after slaughter. The slaughter was carried out in accordance with the recommendations of the Directive of the European Parliament and of the Council of the European Union 2010/63 EC [45], the European Convention for the Protection of Vertebrates, used for experiments and other scientific purposes (ETS No. 123) [46].

On electronic scales, the mass of the animal before slaughter and the mass of the carcass after slaughter were determined with an accuracy of 0.1 kg, and the carcass yield and the slaughter yield were calculated. The mass of internal fat was taken into account. After the mechanical separation of muscle tissue from bones with tendons, the meat content coefficient was determined, expressed by the ratio of the first value to the second. In the carcass’s main topographic areas (hip, lumbar, spinal-scapular), muscle tissue samples of 100 g were taken to perform chemical analysis in a total sample of minced meat. The percentage of moisture, protein, fat, and ash was determined in accordance with accepted national methods [47,48,49]. The area of the loin eye was determined on a paper print obtained when applied to a transverse incision of the longest back muscle. Caloric content was calculated by the formula:

K = [D − (F + A)] × 4.1 + (F × 9.3),

where K is the caloric content, kcal; D, A, F are the content of dry matter, ash, and fat, respectively.

## 3. Results

### 3.1. Population Stratification

**Figure 2:**
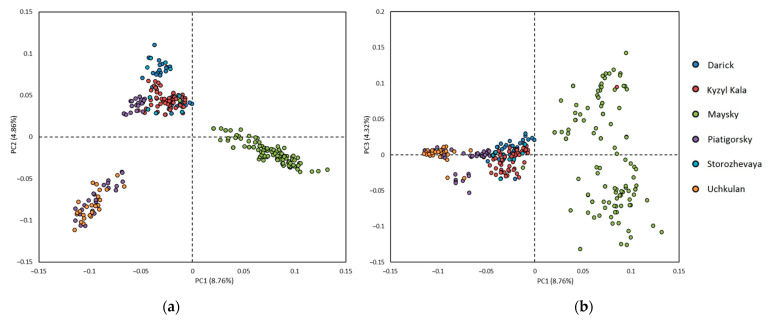
Population structure from the principal component analysis (PCA). PCA plots show the distribution of individuals of Karachai goats in the dimensions of two coordinates, i.e., (**a**) the first (PC1; *X*-axis) and second (PC2; *Y*-axis) principal components, (**b**) the first (PC1; *X*-axis) and third (PC3; *Y*-axis) principal components, with the percentage of total genetic variability, which can be explained by each of the two components, indicated within the parentheses; the individuals from the different herds are indicated by different colors.

The principal component analysis shows a distribution of the studied population between three clusters. The first principal component (PC1), which is responsible for 8.76% of genetic variability, clearly separated the Maysky herd from the five remaining herds, while the principal component two (PC2), which explained 4.86% of genetic variability, distinguished the Darick, Kyzyl Kala and Storozhevaya herds from the Uchkulan herd. The individuals of the Piatigorsky herd were distributed between two clusters (Figure 2).

Considering the observed population stratification, we performed GWAS using the first two PCs as covariates.

### 3.2. Descriptive Statistics

The descriptive GLM statistics for body weight and body conformation traits in Karachai goats at ages four and eight months are summarized in Table 1. Descriptive statistics of the genomic inbreeding coefficient derived from the genomic relationship matrix in Karachai goats by herds revealed a low variation from 3.13 to 12.55% and had a normal distribution for all studied populations.

The growth and development indicators at the age of eight months were characterized by a slightly larger scale of variability compared to similar indicators at four months (Table 1).

### 3.3. Genome-Wide Association Studies

**Figure 3:**
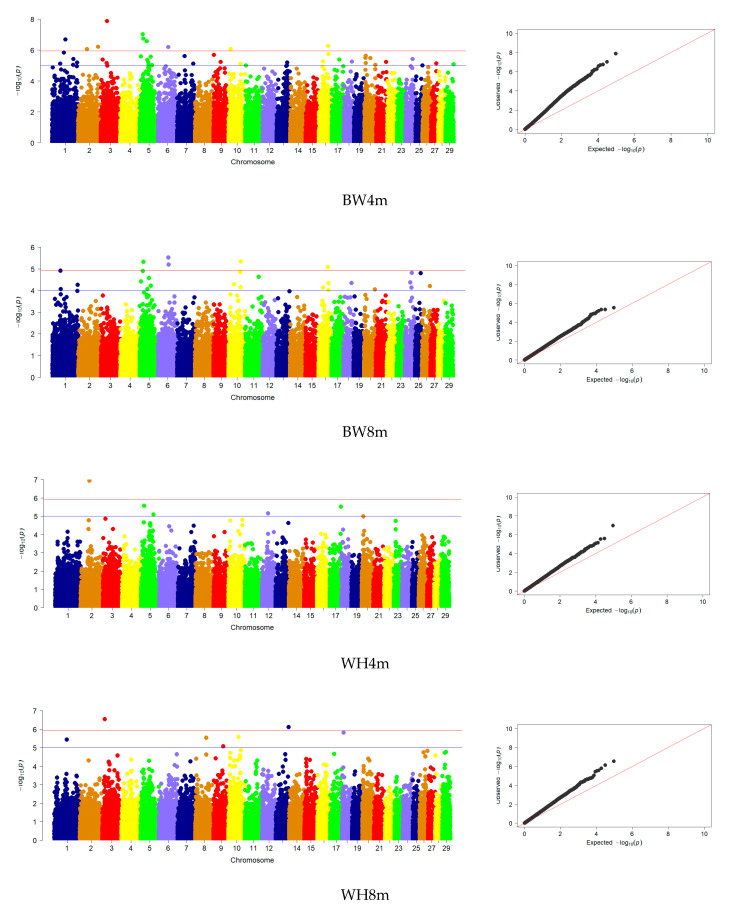

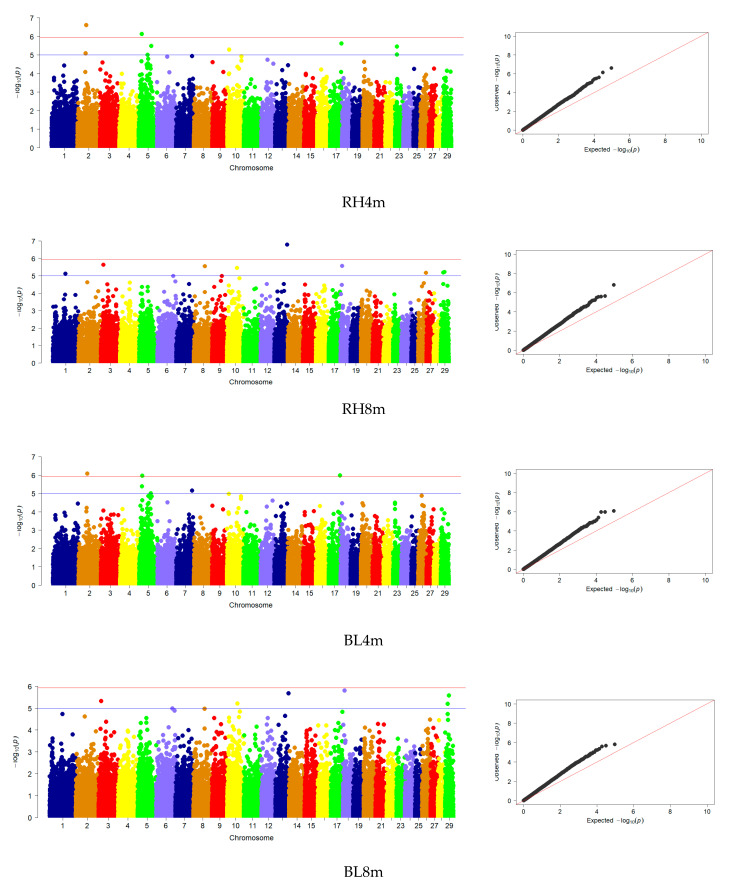

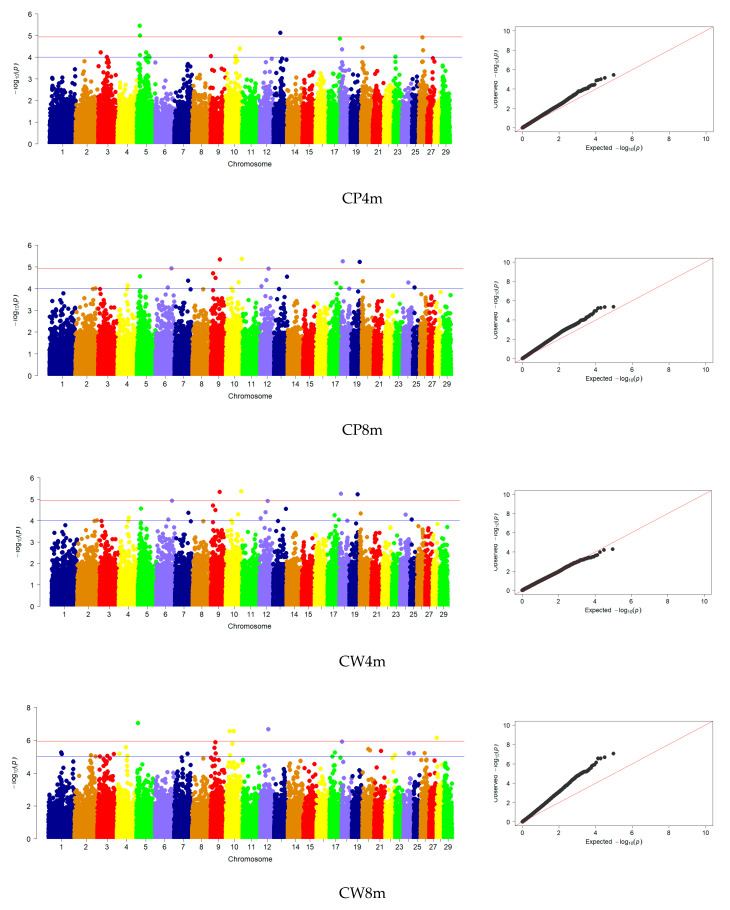

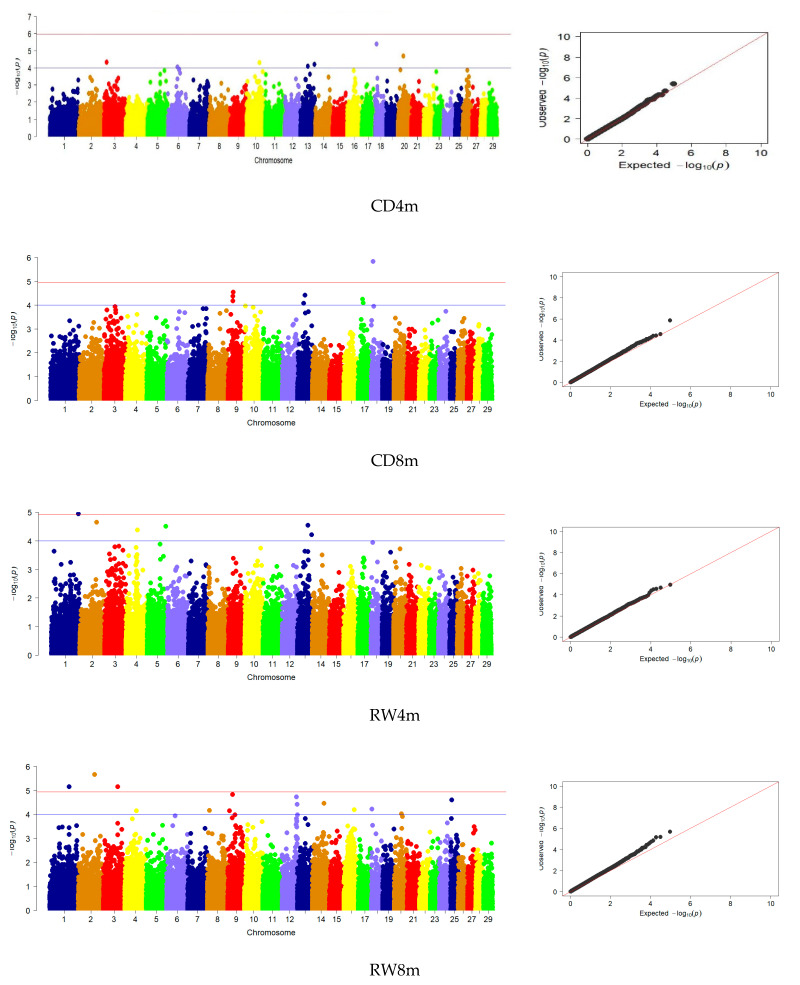
Manhattan plots and QQ plots of the SNPs influencing traits: body weight (**BW**); withers height (**WH**); rump height (**RH**); body length (**BL**); chest perimeter (**CP**); chest width (**CW**); chest depth (**CD**); rump width (**RW**); 4m—4 months, 8m—8 months in Karachai goats using GoatSNP53K Bead Chip.

A comparative analysis of the GWAS results for the actual phenotypic values of the studied traits at the ages of 4 and 8 months showed that for most of them, the observed distribution of deviations from the normal distribution for confidence values approached the expected one (Figure 3).

Association analysis made it possible to identify genome-wide SNPs for eight studied traits at the age of 8 months, including live weight (5 SNP), height at the withers (10 SNP), height at the sacrum (9 SNP), oblique trunk length (6 SNP), chest circumference (4 SNP), chest width (30 SNP), chest depth (8 SNP) and width in rump (14 SNP). For comparison, at four months, genome-wide SNPs were identified for five studied traits, including live weight (33 SNP), height at the withers (2 SNP), height at the sacrum (4 SNP), oblique trunk length (2 SNP) and chest depth at four months (1 SNP) (Figure 3, Table 2).

Comparison of the GWAS results for the studied growth and development indicators of young Karachai goats aged 4 and 8 months revealed the presence of 58 common SNPs for different traits (Table S2). 11 common SNPs were identified for the body weight (BW) trait (snp6325-scaffold1223-530258,snp14251-scaffold157-188734,snp32947-scaffold383-159900,snp33417-scaffold392-2662378,snp945-scaffold1025-1329036,snp30641-scaffold339-3412745,snp2144-scaffold1065-411721,snp8624-scaffold131-2001386, snp16745-scaffold1753-34329,snp1555-scaffold1042-1068020,snp54586-scaffold833-2411812) for both ages, while in nine cases they were the only common of all the studied signs.

For three traits—the height at the withers, the height of the sacrum, and body length (WH, RH, BL) there were four SNPs in total (snp3628-scaffold1113-460458, snp50055-scaffold716-3629742, snp31974-scaffold359-260195, snp31438-frame 348-1638233), for two types—height at the withers, height of the sacrum (W, RH), body length and chest depth (BL and CD) correspond to 2 SNPs (snp15461-scaffold164-717536 and snp50508-scaffold725-933710) and 1 SNP (snp41877-scaffold546-944746).

### 3.4. Candidate Genes

Structural annotation of genome regions using Ensemble web resource within ±0.20 Mb window to identify significant SNPs revealed 288 genes (Table S3).

Functional annotation of 288 identified genes revealed 52 genes with described functions regarding gene ontology (http://geneontology.org/, accessed on 15 November 2022) (Table S4).

The functions of the identified genes are mainly related to the regulation of transcription, angiogenesis, body growth, metabolism of fatty acids and lipids, development of the nervous system, and spermatogenesis.

The following approach was used to select SNP candidates for designing a test system. At the first stage, SNPs common to 2 or more signs of growth and development at the ages of 4 and 8 months were selected, while at least one of the signs should achieve a genome-wide level of reliability of the identified associations. A total of 10 such SNPs were identified (Table S5).

Six SNPs out of ten SNP candidates were associated with the body weight at four and eight months of age in Karachai goats (Table S6). Therefore, these six SNPs were selected for further analysis since body weight determines the meat productivity of animals to a greater extent.

The presence of genes localized within the ±200 kb (0.20 Mb) window was established for three SNPs: T47480416C (rs268269710 A/G) and A23345368G (rs268270492 G/A) on Chr 5 (2 and 1 genes, respectively) and A25854668G (rs268234545 A/G) on Chr 10 (3 genes) (Table S7). These three SNPs were selected as SNP candidates for the construction of a multiplex molecular genetic test system.

Analysis of scientific information sources for the previously described functions of the genes presented in Table S7 showed that they were associated with growth and development rates in animals of other species.

The obtained data served as a justification for the feasibility of developing an experimental multiplex test system for genotyping SNP candidates associated with the live weight of young Karachai goats. A detailed analysis of SNP candidates selected for the design of molecular genetic test systems is presented in Table S8.

Based on the generated list of SNP candidates, three test systems were created to determine the alleles of the following polymorphic loci in the goat genome: rs268234545, rs268270492, and rs268269710. The structural solutions underlying the determination of polymorphisms of candidate genes are presented in Table S9.

Primers and fluorescently labeled probes developed for genotyping polymorphism at rs268234545, rs268270492, and rs268269710 loci are presented in Table S10 (nucleotide sequences are written in the 5′->3′ format).

Validation of test systems was performed by comparing genotyping results on 46 goats obtained using Goat 60K BeadChip and developed test systems. The results of genotyping of all 46 animals obtained using the two above-mentioned methodological approaches completely coincided.

The developed test systems were used for genotyping 35 goats and the subsequent study of post-slaughter signs of meat productivity in carriers of different genotypes. The selected animals had a live weight equal to the average for a group of animals of a certain genotype.

Quantitative and qualitative traits of meat productivity after slaughter were studied in animals of different genotypes at the age of eight months, which are presented in Table S11.

This age is determined because up to this period, the most intensive growth in Karachai goats was observed, and there was the greatest transformation of feed nutrients into productivity, which is known to be effective for meat production [50].

Analysis of the data obtained allowed us to establish that according to SNP rs268269710, the best indicators of meat productivity were characterized by goats of the GG genotype. So, in comparison with AA genotype animals, they had a significant advantage in pre-slaughter and the fresh carcass weight, slaughter yield, boneless meat weight and loin eye area by 8.9%; 13.6%; 4.3% (*p* < 0.05); 10.5% (*p* < 0.01) and 5.2% (*p* < 0.05), respectively.

Comparison of animals of different genotypes in terms of meat productivity by SNP rs268270492 revealed that GG genotype compared to AG genotype had higher pre-slaughter, slaughter yield, slaughter weight, boneless meat weight and loin eye area by 15.8%; 25.7% (*p* < 0.01); 8.4% (*p* < 0.05); 18.3% and 15.7% (*p* < 0.01), respectively.

No significant differences between genotypes were found for SNP rs268234545. According to most indicators, the AA-genotype animals tended to be superior.

It should be noted that the difference in the studied indicators of meat productivity between the compared genotypes according to SNP rs268270492 was more significant than between the genotypes according to SNP rs268269710. It is also necessary to distinguish animals of GG genotypes in these two SNPs by the content of boneless meat, which is the most valuable part of the carcass, which makes keeping these genotypes the most preferable for breeding in terms of obtaining more meat products.

The muscle tissue of young Karachai goats of different genotypes, according to the studied SNPs, had no significant differences in protein, fat, caloric content, moisture, and ash. The meat of young goats of all genotypes at the age of eight months was characterized by high protein content—20.9–22.56%, with low-fat content—7.65–9.62%, which indicates its possible dietary properties. At the same time, according to SNP rs268269710, animals of the GG genotype should be distinguished, which was characterized by the best slaughter performance and ratio of protein to fat in meat from the point of view of dietary nutrition. Thus, in the average sample of minced meat of animals of this genotype, 2.47 g of protein per 1.0 g of fat, whereas in the samples of meat of AA and AG genotypes—2.38 and 2.29 g, respectively. No significant differences were found for other SNPs. The best ratio of protein to fat in meat was found in carriers of the AG genotype in SNP rs268270492 and GG genotype in SNP rs268234545—2.65 and 2.71 g, respectively, whereas in animals of other genotypes in the range—2.33–2.46 g. However, the AG genotype in SNP rs268270492 and the GG genotype in SNP rs268234545 did not show better slaughter parameters (Table S11).

Thus, the identified SNPs within a window of ±200 kb (0.20 Mb) from the *CRADD*, *HMGA2*, *MSRB3*, *FUT8*, *MAX* and *RAB15* genes associated with the pre-slaughter weight are more or less involved in the formation of their meat productivity. We conclude that the most promising for further study and use in the selection of Karachai goats to increase meat productivity is the SNP in rs268269710. The GG genotype is associated with a larger live weight of animals, a larger carcass yield, the content of the boneless part in it, and the ratio of protein and adipose tissue in meat preferred for dietary nutrition.

## 4. Discussion

Recently, numerous studies have been conducted to analyze the genetic architecture of goats, as one of the most common domestic species, well adapted to extreme environmental conditions and possessing phenotypic diversity [51]. In the course of these studies, it was possible to identify some genes associated with a variety of economically important traits of goats, such as down and milk productivity and reproduction [16,52,53,54]. To increase meat productivity and include it in breeding programs for greater production of goat meat, an analysis of associations with live weight and signs of body conformation was carried out, and promising candidate genes were identified [26,30,33,34,35,36].

Further research is likely to be related to the identification of new genes, as well as their selection signatures (SS) and the study of multiple effects on economic and even non-economic traits that are important in the selection of desirable or elimination of undesirable goat genotypes, in the development of breeding programs in goat farming [51]. However, the study of goat genomes is still in its infancy compared to other livestock species. However, progress is being made in performing genome-wide studies, identifying genomic variants and QTL analysis [55,56].

The present study was devoted to searching for candidate genes associated with body measurements and live weight of Karachai goats based on genome-wide associations and developing an experimental PCR-RV test system for genotyping significant SNPs. At the same time, we considered it especially important to identify areas of the genome that would be associated with live weight at four months of age, and they would continue to be associated with this indicator until eight months, i.e., until the optimal age of slaughter and obtaining meat with lower fat content.

There is evidence of significant differences in the genetic structure of the same traits in different age periods. For example, in sheep, the genes associated with body weight at birth, at weaning, at six, 12 and 30 months were different [57,58]. Also, gene differences were noted in beef cattle, which demonstrated a relationship with body weight and average daily growth in different periods of ontogenesis—at six and 12 months [59]. We have observed a similar pattern in our research. Of all the identified SNPs, those different for four and eight months were significantly more than those that coincided. This seems to confirm that such phenotypic traits as the weight and body size of animals at a certain growth phase are regulated by many genes, while these genes may differ or have different expression levels. Therefore, our hypothesis is based on identifying such genes that would demonstrate an association in different periods of growth, and the presence of a functional annotation of such genes was considered mandatory.

For the studied traits at the ages of four and eight months, 58 common SNPs were identified and annotated for 288 genes. Function descriptions in terms of gene ontology are available for 52 of these genes. Despite their significant number, no matches were found with genes for which associations with body size and weight were determined in other studies. At the same time, the generalization of the described functions of candidate genes identified for Karachai goats showed their similarity to those known for other breeds of goats and sheep. Thus, the analysis of ontology and signaling pathways showed that in goats of different breeds, they regulate proliferation, growth, development of cells of the whole organism, and myocytes in particular, cell transport, transcription and translation, reproduction functions, as well as the process of limb development, homeostasis and bone remodeling, collagen synthesis [26,27,28,30,31,32,33,34,35,36]. Candidate genes associated with height at the withers, chest and lower leg girth, body weight gain, loin eye area, and fat thickness are transcription factors that regulate myocyte proliferation and fatty acid metabolism [57,58]. In our study, the main molecular functions of proteins encoded by the identified candidate genes are regulating transcription processes, cell proliferation, angiogenesis, body growth, fatty acid and lipid metabolism, nervous system development, and spermatogenesis.

One of the tasks of our research was to design a test system for genotyping Karachai goats according to the most significant SNPs, the selection of which was carried out in three stages: (1) common SNPs for two or more signs of growth and development at the age of 4 and 8 months, while at least one of the signs should have a genome-wide level of association confidence; (2) then common SNPs for live weight only; and (3) SNPs localized within a window ±200 kb of structural genes. In the first stage, ten were revealed. In the second—six, and the third—three SNPs were identified in the vicinity of six genes CRADD, HMGA2, MSRB3, FUT8, MAX, RAB15, and five of which functions in gene ontology were described.

Thus, it was found that the CRADD gene, along with SOCS2 and PLXNC1 genes, is lo-calized within the so-called “high-growing region” on chromosome 10 in mice. Those genes are associated with the phenotype of high growth in peace and with no obesity [60]. Another study demonstrated a significant relationship between the “high-growing region” mouse genes CRADD, SOCS2 and PLXNC1, as well as two closely located genes, ATP2B1 and DUSP6, with the growth rate in pigs, as well as the quality of meat and fat [61].

Previous studies have revealed a high level of HER2 expression during embryogenesis and a key role in growth-determining and early postnatal development [62]. CRISPR/Cas9 was used in mice to generate an HMGA2 null allele that disrupts only the coding sequence. It was shown that the loss of one or both HMGA2 alleles decreased body weight by 20% and 60%, respectively, compared with wild-type mice from the same litter [63]. The association of the HMGA gene with growth and body weight has also been found in pigs and dogs. It was found that it is activated during early development, participates in the processes of cell division, and the body weight of the animal is highly correlated with HMGA expression [64,65,66]. The MSRB3 gene is an important member of the MSL gene family, which regulates the catalyzing effect of methionine-R-sulfoxide to methionine [67]. In four indigenous cattle breeds selected in China, individual SNPs in the MSRB3 gene were associated with traits of body growth and conformation (body weight, rump height, body length, and chest width) [68,69]. The FUT8 gene encodes an enzyme that belongs to the fucosyltransferase family. Deletion of the FUT8 gene in mice has been shown to lead to growth retardation during postnatal development [70]. The MAX gene encodes MYC-associated factor X (MAX). MAX protein re-lates to the bHLHZ family of transcription factors (Basic helix-loop-helix leucine zipper transcription factors). It forms a transcriptional network with MYC and MAD proteins (MAX-MYC-MAD), which regulates cell proliferation, differentiation, and apoptosis [71].

Sufficiently convincing data on the connection of the above genes with signs of animal growth were the justification for their selection for developing a PCR-RV test system. It was designed, successfully validated, and used for genotyping young goats of the next generation and studying their meat productivity indicators after slaughter. The results showed that the SNP in the rs268269710 position is the most promising for further research and use in breeding Karachai goats. The GG genotype is associated with a larger live weight of animals, increased carcass yield, and the content of the pulp part in it, but also the ratio of protein and adipose tissue in meat preferred for dietary nutrition. The data obtained seem to be explained by the fact that the SNP at the rs268269710 position is located in close proximity to the HMGA2 and MSRB3 genes, for which a more pronounced association with growth energy in young animals has been described [62,63,64,65,66,67,68,69].

Unfortunately, it is not possible to compare our data with the results of other studies. As a rule, such works demonstrate the result of GWAS, functional annotation of genes for identified SNPs, and description of protein products of candidate genes [51,72,73,74]. We have not been able to find studies that would present the results of the PCR tests development and the effectiveness of their use in goat breeding.

In conclusion, we would like to emphasize that our results are preliminary, and to confirm the feasibility of using a PCR test for genotyping and selecting animals preferred for increasing goat meat production, further testing on a larger sample of animals with additional studies on the meat qualities of animals of different genotypes is necessary.

## 5. Conclusions

Thus, for the first time, GWAS of Karachai goats was conducted to identify genes associated with body traits at different ages. We identified 58 common SNPs for different signs of body size (conformation) and body weight at 4 and 8 months. The genes CED, HMGA2, MSRB3, FUT8, MAX, and RUBI 15 are candidates for signs of body weight and meat productivity and are promising for further priority studies. These results will make a significant contribution to understanding the mechanisms underlying the signs of meat productivity and will contribute to the genetic improvement of Karachai goats.

## Figures and Tables

**Table 1:** Descriptive GLM statistics for body weight and body conformation traits in Karachai goats at age four and eight months.

Trait	Max	Min	Mean	Var	Std. Dev	CV %
Four months						
BW	36.5 kg	19.1 kg	24.75 kg	15.58	3.94	14.54
WH	53.0 cm	45.5 cm	48.97 cm	3.25	1.80	3.72
RH	54.5 cm	46.5 cm	49.85 cm	3.49	1.87	3.81
BL	56.0 cm	47.5 cm	51.00 cm	3.55	1.89	3.70
CP	57.5 cm	49.5 cm	53.12 cm	2.71	1.65	3.13
CW	12.0 cm	7.5 cm	8.98 cm	0.57	0.75	8.44
CD	23.5 cm	17.5 cm	19.78 cm	1.53	1.23	6.35
RW	12.0 cm	9.5 cm	10,28 cm	0.20	0.45	4.42
Eight months						
BW	49.8 kg	27.1 kg	36.35 kg	14.8	4.21	11.59
WH	60.5 cm	47.5 cm	52.78 cm	8.11	2.84	5.38
RH	60.5 cm	48.0 cm	53.48 cm	8.95	2.99	5.59
BL	61.0 cm	48.1 cm	54.12 cm	9.15	3.02	5.58
CP	69.0 cm	52.2 cm	60.60 cm	10.96	3.31	5.46
CW	14.0 cm	8.0 cm	10.21 cm	1.64	1.28	12.55
CD	26.5 cm	18.0 cm	21.54 cm	2.91	1.71	7.95
RW	13.9 cm	9.5 cm	11.30 cm	0.67	0.82	6.86

BW (body weight), WH (withers height), RH (rump height), BL (body length), CP (chest perimeter), CW (chest width), CD (chest depth), RW (rump width), Var: variation, Std. Dev: standard deviation, CV: coefficient of variation.

**Table 2:** Number and chromosome distribution of significant (*p* < 10^−5^) SNPs associated with body weight and body conformation traits at 4 and 8 months of age.

Trait	8 Months		4 Months	
	n	Chr	n	Chr
BW	5	5, 6, 10, 16	33	1, 2, 3, 5, 6, 7, 9, 10, 13, 16, 17, 20, 24, 26
WH	10	1, 3, 8, 9, 10, 13, 18	2	2, 20
RH	9	1, 3, 8, 10, 13, 18, 26, 29	4	2, 5, 20, 23
BL	6	3, 10, 13, 18, 29	2	2, 5
CP	4	9, 10, 18, 19	-	-
CW	30	1, 2, 3, 4, 5, 7, 9, 10, 12, 17, 18, 20, 21, 24, 26, 28	-	-
CD	8	9, 13, 17, 18	1	18
RW	14	1, 2, 3, 4, 8, 9, 12, 14, 16, 18, 20, 25	-	-

BW (body weight), WH (withers height), RH (rump height), BL (body length), CP (chest perimeter), CW (chest width), CD (chest depth), RW (rump width).

## Data Availability

Not applicable.

## Supplementary Materials

The supporting information can be downloaded at https://www.mdpi.com/article/10.3390/ani13203237/s1; Table S1. Body measurements in yang goats at 4 and 8 months; Table S2 List of common SNPs associated with body conformation traits at both 4 and 8 months of age in Karachai goats; Table S3: Closest candidate genes for genome-wide SNPs (*p* < 10^−5^) associated with body weight and body conformation traits based on GWAS in Karachai goats; Table S4: Functional annotation of candidate genes; Table S5: SNP candidates selected on the results of GWAS, common for two or more signs of growth and development at the age of 4 and 8 months; Table S6: Candidate SNPs associated with the body weight at 4 and 8 months of age in Karachai goats; Table S7: SNP candidates associated with the live weight of Karachai goats used to construct a molecular genetic test system; Table S8: SNP candidates analysis for the molecular genetic test systems design; Table S9: Modeling the definition of polymorphism in position: (a) rs268270492; (b) rs268234545; (c) rs268269710; Table S10: Primers and fluorescent probes developed for genotyping polymorphism in loci rs268270492, rs268234545, rs268269710; Table S11: Indicators of meat productivity of young Karachai goats of different genotypes by SNP rs268269710, rs268270492, rs268234545 (8 months). The SNP genotypes of 269 Karachai goats used for genome-wide association studies are available in the ZENODO repository (https://doi.org/10.5281/zenodo.7053677, Uploaded on 6 September 2022).
