# Supplementary material for: Genome-Wide Association and Pathway Analysis of Carcass and Meat Quality Traits in Karachai Young Goats
Source: Animals (Basel). 2023 Oct 17;13(20):3237. doi: 10.3390/ani13203237 (PMC10603756; doi:10.3390/ani13203237)
Supplement: Supplementary file 1 [file animals-13-03237-s001.zip › animals-2558096-supplementary.pdf]

Table S1. Body measurements in yang goats at four and eight months

"Darik"				"Uchkulan"				"Storozhevaya"			
Four months				Four months				Four months			
**Trait**	**Mean**	**Std. Dev**	**CV,%**	**Trait**	**Mean**	**Std. Dev**	**CV,%**	**Trait**	**Mean**	**Std. Dev**	**CV,%**
BW, kg	24,17	3,19	17,01%	BW, kg	23	3,06	18,82%	BW, kg	22,79	3,41	11,42%
WH, cm	49,33	1,8	3,24%	WH, cm	48,8	1,46	2,13%	WH, cm	49,36	1,97	3,82%
RH, cm	50,23	1,81	3,29%	RH, cm	49,66	1,48	2,20%	RH, cm	50,32	1,98	3,85%
BL, cm	51,36	1,73	3,00%	BL, cm	50,78	1,67	2,79%	BL, cm	51,46	2,13	4,34%
CP, cm	53,33	1,5	2,25%	CP, cm	52,8	1,23	1,52%	CP, cm	53,21	1,58	2,51%
CW, cm	8,84	0,77	0,59%	CW, cm	8,86	0,68	0,47%	CW, cm	9	0,76	0,55%
CD, cm	19,94	1,41	1,99%	CD, cm	19,66	1,19	1,41%	CD, cm	19,82	1,23	1,46%
RW, cm	10,44	0,55	0,30%	RW, cm	10,42	0,45	0,20%	RW, cm	10,32	0,46	0,21%
Eight months				Eight months				Eight months			
**Trait**	**Mean**	**Std. Dev**	**CV,%**	**Trait**	**Mean**	**Std. Dev**	**CV,%**	**Trait**	**Mean**	**Std. Dev**	**CV,%**
BW, kg	37,47	3,95	15,58%	BW, kg	36,38	4,1	16,84%	BW, kg	36,57	3,97	15,80%
WH, cm	55,08	2,74	7,50%	WH, cm	52,94	2,44	5,96%	WH, cm	53,32	2,56	6,56%
RH, cm	55,9	2,58	6,67%	RH, cm	53,58	2,5	6,26%	RH, cm	53,96	2,87	8,21%
BL, cm	56,84	2,56	6,56%	BL, cm	54,1	2,42	5,83%	BL, cm	54,79	3,04	9,22%
CP, cm	62,21	2,82	7,98%	CP, cm	61,06	2,81	7,88%	CP, cm	61,75	3,41	11,60%
CW, cm	12,13	0,87	0,75%	CW, cm	9,72	0,78	0,61%	CW, cm	9,93	1,04	1,07%
CD, cm	21,98	1,63	2,64%	CD, cm	21,64	1,63	2,66%	CD, cm	21,25	1,38	1,91%
RW, cm	11,94	0,86	0,75%	RW, cm	11,56	0,6	0,66%	RW, cm	11,39	0,81	0,66%
**"Kyzyl-Kala"**				**"Pyatigorsky"**				**"Maysky"**			

Four months				Four months				Four months			
**Trait**	**Mean**	**Std. Dev**	**CV,%**	**Trait**	**Mean**	**Std. Dev**	**CV,%**	**Trait**	**Mean**	**Std. Dev**	**CV,%**
BW, kg	23,12	2,61	6,82%	BW, kg	24,37	2,98	9,89%	BW, kg	23,92	2,95	7,39%
WH, cm	48,39	1,44	2,07%	WH, cm	49,06	1,67	4,46%	WH, cm	49,15	2,03	3,76%
RH, cm	49,22	1,53	2,34%	RH, cm	50	1,73	7,08%	RH, cm	50	2,11	4,01%
BL, cm	50,39	1,5	2,25%	BL, cm	51,17	1,77	6,26%	BL, cm	51,15	2,11	4,00%
CP, cm	52,95	1,61	2,58%	CP, cm	53,24	1,43	6,58%	CP, cm	53,19	1,88	3,24%
CW, cm	9,04	0,67	0,45%	CW, cm	8,89	0,78	1,36%	CW, cm	9,08	0,8	0,65%
CD, cm	19,63	1,17	1,37%	CD, cm	19,72	1,24	2,30%	CD, cm	19,87	1,25	1,56%
RW, cm	10,18	0,42	0,17%	RW, cm	10,3	0,48	0,44%	RW, cm	10,26	0,41	0,19%
Eight months				Eight months				Eight months			
**Trait**	**Mean**	**Std. Dev**	**CV,%**	**Trait**	**Mean**	**Std. Dev**	**CV,%**	**Trait**	**Mean**	**Std. Dev**	**CV,%**
BW, kg	35,08	3,65	13,29%	BW, kg	36,91	3,37	9,13%	BW, kg	36,1	3,86	13,52%
WH, cm	52,04	2,51	6,29%	WH, cm	52,73	2,49	4,44%	WH, cm	52,37	3	8,98%
RH, cm	52,65	2,8	7,84%	RH, cm	53,42	3,03	4,96%	RH, cm	53,11	2,99	8,94%
BL, cm	53,53	3,02	9,15%	BL, cm	54,14	2,87	4,79%	BL, cm	53,58	3,03	9,09%
CP, cm	60,14	3,92	15,40%	CP, cm	60,96	3,17	4,78%	CP, cm	59,93	3,2	9,88%
CW, cm	10,15	1,13	1,27%	CW, cm	9,87	1,3	0,79%	CW, cm	9,95	1,08	1,13%
CD, cm	21,27	1,95	3,79%	CD, cm	21,66	1,72	2,30%	CD, cm	21,42	1,67	2,79%
RW, cm	11,16	0,84	0,70%	RW, cm	11,19	0,73	0,32%	RW, cm	11,15	0,79	0,56%

Table S2 List of common SNPs associated with body conformation traits at both 4 and 8 months of age in Karachai goats.

	SNP	Position		Trait
		AdaptMap	ARS1.2	
1	snp29553-scaffold320-1261495	147 219 943	149 596 828	BW4m, WH4m, CW8m
	snp36273-scaffold435-778554	75 496 238	76 791 560	BW8m, BL4m
	snp37579-scaffold46-3395473	149 924 303	152 300 137	BW4m, CW8m
	snp48335-scaffold686-556322	46 866 236	47 955 872	BW4m, RH8m
2	snp3628-scaffold1113-460458	119 046 372	16 103 527	BW4m, WH4m, WH8m, RH4m, RH8m, BL4m, BL8m
	snp45045-scaffold614-2989486	48 583 647	87 021 794	BW4m, CW8m
	snp45128-scaffold614-6524265	52 118 426	83 474 726	BL8m, RH8m, WH8m
	snp45987-scaffold631-242453	94 055 097	41 146 618	BW4m, CW8m
3	snp15461-scaffold164-717536	44 049 812	74 329 119	BW4m,WH4m, WH8m, BL8m, RH4m, RH8m, CW8m, CD4m
4	snp24555-scaffold2498-324452	15 657 299	103 535 122	BW4m, WH8m,
	snp43974-scaffold595-3225253	92 070 125	25 235 510	BW4m, BL8m
	snp6325-scaffold1223-530258	11 881 912	107 364 229	BW4m, BW8m
5	snp14251-scaffold157-188734	19 499 085	19 934 148	BW4m, BW8m
	snp28866-scaffold310-6866081	68 925 209	69 743 011	BW4m, WH8m, RH8m, BL8m
	snp32947-scaffold383-159900	69 928 227	70 744 300	BW4m, BW8m
	snp33373-scaffold392-511730	56 209 048	57 098 635	BW4m, BW8m, CW8m
	snp33417-scaffold392-2662378	54 058 400	54 952 435	BW4m, BW8m
	snp33463-scaffold392-4664782	52 055 996	52 930 625	BW4m, CW8m
6	snp11972-scaffold1440-170733	109 087 772	112 296 759	BW4m, WH8m, RH8m, BL8m, CP8m
	snp2183-scaffold1066-1111590	73 165 497	75 620 791	BW4m, CW8m

	SNP	Position		Trait
		AdaptMap	ARS1.2	
	snp40053-scaffold511-1070977	61 826 989	64 228 064	BW4m, WH8m, CD8m
	snp40083-scaffold511-2344051	60 553 915	62 949 329	BW4m, CW8m
	snp945-scaffold1025-1329036	95 725 920	99 053 758	BW4m, BW8m
7	snp30641-scaffold339-3412745	44 814 083	61 869 256	BW4m, BW8m, WH8m, RH8m, BL8m, CP8m
	snp4684-scaffold1152-350655	96 054 404	10 521 909	RH8m, WH8m, BL8m, CP8m
	snp50055-scaffold716-3629742	76 765 612	29 871 014	BW4m, WH4m, WH8m, RH4m, RH8m, BL4m, BL8m
8	snp12947-scaffold1499-2414292	66 113 241	67 503 568	BL4m, BW8m
9	snp10101-scaffold1358-1351350	27 789 793	27 999 512	BW4m, CW8m
	snp29395-scaffold318-901939	52 868 428	53 346 107	BW4m, RH8m
	snp31974-scaffold359-260195	12 795 958	12 886 633	BW4m, WH4m, WH8m, RH4m, RH8m, BL4m, BL8m
	snp3575-scaffold1110-924176	58 547 070	59 047 836	BW4m, CW8m
	snp39588-scaffold504-358366	2 394 396	2 388 136	WH8m, RH8m, BL8m
10	snp40816-scaffold524-739363	25 782 240	25 989 680	BW4m, CW8m
	snp50508-scaffold725-933710	66 049 702	66 584 485	BW4m, WH4m, WH8m, RH4m, RH8m, BL8m, CW8m, CD4m
11	snp1481-scaffold104-2612843	71 882 233	27 289 223	BW4m, WH8m
	snp1502-scaffold104-3550558	70 944 518	28 278 268	BW4m, BL8m
	snp2144-scaffold1065-411721	74 906 896	24 296 772	BW4m, BW8m
	snp7931-scaffold1287-749597	69 635 073	29 620 817	CD4m, WH8m, CW8m
12	snp23209-scaffold2318-21647	41 071 978	41 908 344	CD4m, CD8m, CP4m
	snp31438-scaffold348-1638233	76 566 792	77 996 024	BW4m, WH4m, WH8m, RH4m, RH8m, BL4m, BL8m, CP8m, CD4m, RW4m
	snp48699-scaffold691-2724642	57 786 919	59 166 263	BW4m, BL8m, WH8m, RH8m, CW8m

	SNP	Position		Trait
		AdaptMap	ARS1.2	
	snp48703-scaffold691-2873716	57 637 845	59 016 707	BW4m, CW8m
13	snp59936-scaffold998-300033	50 269 992	42 374 922	BW4m, CW8m
14	snp17224-scaffold18-62949	24 094 863	24 898 037	BW4m, CW8m
	snp3682-scaffold112-1043886	59 461 662	60 447 085	BW4m, BW8m
	snp3684-scaffold112-1122107	59 539 883	60 525 389	BW4, BW8m
	snp3953-scaffold1125-190766	39 246 435	40 120 683	BW4m, WH8m, RH8m
	snp8624-scaffold131-2001386	56 416 291	57 408 452	BW4m, BW8m
15	snp16280-scaffold1714-321063	43 968 904	26 782 979	BW4m, BW8m
	snp35564-scaffold428-2794282	31 865 532	38 994 171	CW8m, BW4m
16	snp19484-scaffold1967-768885	47 641 690	49 693 270	BW4m, BW8m
	snp41877-scaffold546-944746	9 951 450	11 898 552	WH8m, RH8m, BL4m, BL8m, CP8m, CW8m, CD4m, CD8m
17	snp16745-scaffold1753-34329	66 174 065	66 737 512	BW4m, BW8m
	snp52283-scaffold776-291202	36 434 931	36 698 503	BW4m, WH8m, RH8m CW8m,
18	snp1555-scaffold1042-1068020	32 996 397	33 188 152	BW4m, BW8m, CW8m
	snp54586-scaffold833-2411812	41 158 871	41 365 838	BW8m, BW4m
19	snp47533-scaffold67-1525470	34 443 948	15 957 679	CW8m, BW4m

Table S3 Closest candidate genes for genome-wide SNPs ($p < 10^{-5}$) associated with body weight and body conformation traits based on GWAS in Karachai goats

Chr	Position *	SNP	Genes **
1	149 596 828	snp29553-scaffold320-1261495	*DYRK1A*$^{149441823..149586835}$, *KCNJ6*$^{149701472..149797540}$
1	76 791 560	snp36273-scaffold435-778554	*CLDN16*$^{76623425..76647770}$, *CLDN1*$^{76761492..76779219}$, *P3H2*$^{76910759..77089601}$
1	152 300 137	snp37579-scaffold46-3395473	***EAF1***$^{152290977..152305196}$, *SH3BP5*$^{152126639..152198013}$, *CAPN7*$^{152074727..152125699}$, *METTL6*$^{149899417..149915521}$, *BTD*$^{152470196..152530009}$, *HACL1*$^{152427023..152470636}$, *COLQ*$^{152314010..152379414}$
1	80 175 337	snp40557-scaffold519-2601256	*ST6GAL1*$^{79880646..80020253}$, *ADIPOQ*$^{80092745..80105787}$, *RFC4*$^{80128438..80141956}$, *EIF4A2*$^{80141693..80148253}$, *MIR1248*$^{80144820..80144924}$, *KNG1*$^{80175447..80201003}$, *TBCCD1*$^{80319786..80347985}$, *DNAJB11*$^{80303639..80323058}$, *CRYGS*$^{80347993..80353625}$, *FETUB*$^{80226559..80241470}$, *AHSG*$^{80273479..80280715}$, *HRG*$^{80206861..80214843}$
2	16 103 527	snp3628-scaffold1113-460458	***DIS3L2***$^{15749236..16107599}$, *NPPC*$^{16144029..16148670}$, *COPS7B*$^{16210787..16236131}$, *PDE6D*$^{16236483..16290580}$
2	83 474 726	snp45128-scaffold614-6524265	*ARHGAP15*$^{82654446..83365942}$, *GTDC1*$^{83524315..83969888}$

Chr	Position *	SNP	Genes **
2	41 146 618	snp45987-scaffold631-242453	***ADAM23***$^{41016764..41207167}$, *DYTN*$^{40922895..40981626}$, *ZDBF2*$^{41304927..41315247}$
3	74 329 119	snp15461-scaffold164-717536	***DPYD***$^{74140748..75067664}$
3	80 835 937	snp22453-scaffold222-851829	*RNPC3*$^{80656949..80688529}$
3	103 535 122	snp24555-scaffold2498-324452	*INTS3*$^{103488703..103528260}$, *SLC27A3*$^{103529170..103534260}$, *NPR1*$^{103453176..103467036}$, *CHTOP*$^{103403168..103412805}$, *ILF2*$^{103435735..103442739}$, *SNAPIN*$^{103434079..103435783}$, *NUP210L*$^{103700807..103790482}$, *DENND4B*$^{103646814..103661156}$, *RAB13*$^{103691871..103699082}$, *GATAD2B*$^{103563417..103643132}$, *CREB3L4*$^{103679930..103685385}$, *CRTC2*$^{103662689..103672394}$, *JTB*$^{103685754..103688387}$, *RPS27*$^{103699112..103700692}$
3	25 235 510	snp43974-scaffold595-3225253	***FAF1***$^{24994347..25472808}$
3	39 664 309	snp48395-scaffold687-2186794	***UBE2U***$^{39591365..39669695}$**,** *ROR1*$^{39115242..39572757}$
3	107 364 229	snp6325-scaffold1223-530258	***FCRL3***$^{107353691..107414661}$**,** *FCRL5*$^{107178895..107253732}$, *FCRL4*$^{107264514..107288861}$, *FCRL1*$^{107426215..107442566}$, *CD5L*$^{107533545..107548435}$
4	59 721 313	snp15777-scaffold1654-1487108	*AOAH*$^{59458372..59656794}$, *ELMO1*$^{59752190..60332615}$
5	67 987 054	snp28824-scaffold310-5114951	*NUAK1*$^{68166557..68240209}$
5	69 743 011	snp28866-scaffold310-6866081	***BPIFC***$^{69725751..69770627}$, *ABTB3*$^{69267173..69589324}$,

Chr	Position *	SNP	Genes **
			PRDM4$^{69650821..69675377}$,<br>*PWP1*$^{69612078..69630949}$,<br>*SYN3*$^{69807334..70282973}$,<br>*FBXO7*$^{69782359..69802083}$,<br>*RTCB*$^{69696203..69723193}$,<br>*ASCL4*$^{69689101..69690926}$
5	76 155 531	snp32361-scaffold368-35564	***BICD1***$^{76054530..76298702}$,<br>*FGD4*$^{75790760..76027336}$
5	70 744 300	snp32947-scaffold383-159900	***LARGE1***$^{70481063..71091017}$
5	57 098 635	snp33373-scaffold392-511730	*METTL7B*$^{56935391..56937889}$,<br>*BLOC1S1*$^{56901902..56905728}$
5	54 952 435	snp33417-scaffold392-2662378	*CTDSP2*$^{54975054..54998111}$,<br>*ATP23*$^{54860513..54880033}$,<br>*METTL1*$^{55035256..55039587}$,<br>*OS9*$^{55087193..55122775}$,<br>*AGAP2*$^{55063063..55080580}$,<br>*AVIL*$^{54998163..55017814}$,<br>*TSFM*$^{55018794..55026954}$,<br>*EEF1AKMT3*$^{55027142..55034769}$,<br>*MARCHF9*$^{55047130..55051800}$,<br>*CDK4*$^{55055732..55058821}$,<br>*TSPAN31*$^{55058825..55061830}$
5	53 138 546	snp33458-scaffold392-4457951	*SLC16A7*$^{52967562..53161674}$
5	52 930 625	snp33463-scaffold392-4664782	*SLC16A7*$^{52967562..53161674}$
5	47 480 416	snp37630-scaffold463-64670	*MSRB3*$^{47675346..47862677}$,<br>*HMGA2*$^{47162168..47306445}$
5	23 345 368	snp38426-scaffold486-2412676	***CRADD***$^{23232979..23425649}$
5	8 501 612	snp54059-scaffold822-1742056	***SYT1***$^{8236497..8861733}$
6	112 296 759	snp11972-scaffold1440-170733	***LDB2***$^{111976958..112438346}$
7	61 869 256	snp30641-scaffold339-3412745	***SPOCK1***$^{61566246..62155271}$
7	10 521 909	snp4684-scaffold1152-350655	***ST8SIA4***$^{10496801..10602994}$

Chr	Position *	SNP	Genes **
7	29 871 014	snp50055-scaffold716-3629742	*ANKRD34B*$^{29871879..29887615}$, *MSH3*$^{29624244..29815981}$, *DHFR*$^{29816045..29831001}$, *FAM151B*$^{29900379..29933088}$, *RARS1*$^{30037187..30065836}$
9	27 999 512	snp10101-scaffold1358-1351350	***ZBTB24***$^{\mathbf{27989453..28000976}}$, *FIG4*$^{27692407..27859852}$, *PPIL6*$^{28022791..28059970}$, *AK9*$^{27860458..27981818}$, *MICAL1*$^{28007621..28019243}$, *CD164*$^{28069710..28081880}$, *SMPD2*$^{28019378..28022352}$
9	53 346 107	snp29395-scaffold318-901939	***PTPRK***$^{\mathbf{53315015..53933095}}$, *THEMIS*$^{53018391..53225409}$
9	12 886 633	snp31974-scaffold359-260195	*NCOA7*$^{12608402..12765566}$, *HEY2*$^{12796333..12808691}$
9	59 047 836	snp3575-scaffold1110-924176	*EYA4*$^{58526657..58849760}$, *TBPL1*$^{59202000..59242065}$, *TCF21*$^{59157639..59160660}$
9	2 388 136	snp39588-scaffold504-358366	*FILIP1*$^{2133407..2386513}$, *SENP6*$^{2454528..2585888}$
9	25 989 680	snp40816-scaffold524-739363	***FYN***$^{\mathbf{25894431..26111694}}$, *TRAF3IP2*$^{26149367..26194655}$
9	66 584 485	snp50508-scaffold725-933710	*VTA1*$^{66664723..66737542}$, *NMBR*$^{66613447..66622175}$
10	25 854 668	snp1448-scaffold104-1147808	*MAX*$^{25900233..25924465}$, *RAB15*$^{26026720..26050417}$, *GPX2*$^{26053403..26056892}$
10	27 289 223	snp1481-scaffold104-2612843	*PPP2R5E*$^{27321023..27477743}$, *DR89*$^{27228084..27276570}$, *SGPP1*$^{72011216..72053701}$

Chr	Position *	SNP	Genes **
10	66 185 170	snp16064-scaffold1690-412631	***RTF1***$^{66154961..66200148}$,<br>*MGA*$^{65893435..66043984}$,<br>*TYRO3*$^{66086217..66103879}$,<br>*RPAP1*$^{66111130..66127784}$,<br>*LTK*$^{66129428..66137798}$,<br>*ITPKA*$^{66138462..66146811}$,<br>*NDUFAF1*$^{66227630..66247703}$,<br>*EXD1*$^{66350192..66388591}$,<br>*CHP1*$^{66313916..66349778}$,<br>*NUSAP1*$^{66249743..66279326}$,<br>*OIP5*$^{33155842..33167160}$
10	24 296 772	snp2144-scaffold1065-411721	***GPHN***$^{24055825..24607616}$,
10	90 722 528	snp49271-scaffold703-2391474	*HOMER1*$^{90737422..90876268}$,<br>*JMY*$^{90890185..90973599}$,<br>*CMYA5*$^{90503815..90602388}$,<br>*TENT2*$^{90604800..90668620}$
10	89 474 281	snp49301-scaffold703-3633491	*ANKDD1A*$^{89383639..89425851}$,<br>*SPG21*$^{89345119..89371062}$,<br>*PTGER2*$^{89435648..89446090}$,<br>*MTFMT*$^{89310760..89334973}$,<br>*RASL12*$^{89261527..89277906}$,<br>*SLC51B*$^{89277857..89288048}$,<br>*TXNDC16*$^{89558229..89658168}$,<br>*GPR137C*$^{89658741..89714098}$
10	29 620 817	snp7931-scaffold1287-749597	***PRKCH***$^{29384010..29745599}$,<br>*TMEM30B*$^{29784967..29788468}$
11	67 255 733	snp17962-scaffold185-6628584	***ANTXR1***$^{67083768..67344176}$,<br>*GFPT1*$^{67434693..67501259}$
11	77 656 443	snp20768-scaffold204-3792524	*APOB*$^{77590208..77630122}$,<br>*TDRD15*$^{77503856..77510131}$,<br>*LDAH*$^{77798691..77896694}$

Chr	Position *	SNP	Genes **
12	9 948 039	snp13622-scaffold1526-998265	***TM9SF2***$^{9916049..9966298}$,<br>*CLYBL*$^{9645150..9878794}$,<br>*UBAC2*$^{10054141..10225872}$
13	52 152 409	snp13094-scaffold150-5171386	***TGM3*** $^{52142060..52186060}$,<br>*STK35*$^{52326090..52368178}$,<br>*TGM6*$^{52042714..52078772}$,<br>*SNRPB*$^{52011858..52020500}$,<br>*TMC2*$^{51886958..51986741}$
13	41 908 344	snp23209-scaffold2318-21647	*SYNDIG1*$^{41768426..41877740}$,<br>*ACSS1*$^{41975280..42019317}$,<br>*APMAP*$^{41943197..41969801}$,<br>*CST7*$^{41932208..41942140}$,<br>*ENTPD6*$^{42083119..42100859}$,<br>*VSX1*$^{42035818..42044099}$
13	77 996 024	snp31438-scaffold348-1638233	*PTPN1*$^{78052477..78117075}$,<br>*RIPOR3*$^{78118187..78191610}$
13	18 377 009	snp46284-scaffold638-1700219	*PARD3*$^{17803932..18377838}$
13	59 534 025	snp48690-scaffold691-2355185	***ANGPT4***$^{59493311..59543214}$,<br>*RSPO4*$^{59419545..59456637}$,<br>*FAM110A*$^{59563554..59575513}$,<br>*SCRT2*$^{59693358..59707360}$,<br>*SRXN1*$^{59717581..59724941}$
13	59 166 263	snp48699-scaffold691-2724642	***RAD21L1***$^{59165671..59201265}$,<br>*TMEM74B*$^{59244717..59249746}$,<br>*PSMF1*$^{59259919..59302429}$,<br>*SNPH*$^{59109662..59153612}$,<br>*SDCBP2*$^{59090093..59109110}$,<br>*FKBP1A*$^{59038273..59061754}$,<br>*NSFL1C*$^{59000052..59021299}$
13	59 016 707	snp48703-scaffold691-2873716	*RAD21L1*$^{59165671..59201265}$,<br>*TMEM74B*$^{59244717..59249746}$,<br>*PSMF1*$^{59259919..59302429}$,

Chr	Position *	SNP	Genes **
			SNPH$^{59109662..59153612}$,<br>*SDCBP2*$^{59090093..59109110}$,<br>*FKBP1A*$^{59038273..59061754}$,<br>***NSFL1C***$^{\mathbf{59000052..59021299}}$,<br>*CASS4*$^{58791766..58829365}$,<br>*CSTF1*$^{58833053..58845722}$,<br>*AURKA*$^{58845806..58862526}$,<br>*FAM210B*$^{58864336..58875132}$,<br>*MC3R*$^{58935095..58936185}$
15	47 831 648	snp36734-scaffold445-2068057	***SERGEF***$^{\mathbf{47598321..47853505}}$,<br>*SAAL1*$^{47910630..47936036}$,<br>*TPH1*$^{47860142..47897105}$
16	24 898 037	snp17224-scaffold18-62949	*BROX*$^{24708745..24728139}$,<br>*AIDA*$^{24660030..24708676}$,<br>***DISP1***$^{\mathbf{24799660..25020285}}$
16	26 376 843	snp17258-scaffold18-1536295	*CNIH3*$^{26233683..26368158}$
16	50 997 497	snp30143-scaffold331-984668	*KAZN*$^{50864778..51392076}$,<br>*TMEM51*$^{50763811..50819829}$
16	60 447 085	snp3682-scaffold112-1043886	***XPR1***$^{\mathbf{60399354..60607780}}$,<br>*KIAA1614*$^{60625913..60669842}$,<br>*ACBD6*$^{60059200..60264783}$
16	60 525 389	snp3684-scaffold112-1122107	***XPR1***$^{\mathbf{60399354..60607780}}$,<br>*KIAA1614*$^{60625913..60669842}$,<br>*STX6*$^{60683297..60727907}$
16	40 120 683	snp3953-scaffold1125-190766	*VPS13D* $^{39694426..39961284}$,<br>*PLOD1*$^{40203194..40232944}$,<br>*MFN2*$^{40168595..40195881}$,<br>*MIIP*$^{40142172..40156146}$,<br>*KIAA2013*$^{40238540..40244293}$,<br>*NPPB*$^{40311522..40312961}$
16	56 893 479	snp8636-scaffold131-2516395	***ASTN1***$^{\mathbf{56601271..56960443}}$,<br>*BRINP2*$^{56967843..57112277}$

Chr	Position *	SNP	Genes **
17	26 782 979	snp16280-scaffold1714-321063	***GOLGA3***$^{\mathbf{26739013..26785475}}$,<br>*BRSL1*$^{26509590..26593609}$,<br>*P2RX2*$^{26626197..26629842}$,<br>*POLE*$^{26631093..26687394}$,<br>*PXMP2*$^{26687503..26697725}$,<br>*LRCOL1*$^{26602142..26609535}$,<br>*PGAM5*$^{26699796..26708294}$,<br>*ANKLE2*$^{26708092..26732186}$,<br>*CHFR*$^{26799009..26823758}$,<br>*MBD3L1*$^{26881001..26881552}$
17	3 009 675	snp25671-scaffold264-3189431	*TTC28* $^{3122634..3697004}$,<br>*HSCB*$^{3074644..3083643}$,<br>*CHEK2*$^{3083718..3121516}$,<br>*CCDC117*$^{3049418..3060253}$,<br>*XBP1*$^{3041839..3046873}$,<br>*KREMEN1*$^{2789473..2843831}$
18	49 693 270	snp19484-scaffold1967-768885	*SIRT*$^{49484016..49503706}$,<br>*MRPS12*$^{49529591..49531638}$,<br>*SARS2*$^{49519987..49529496}$,<br>*CCER2*$^{49514933..49517723}$,<br>*FBXO17*$^{49533372..49565201}$,<br>*ACP7*$^{49709295..49725396}$,<br>*PAK4*$^{49768383..49785625}$,<br>*SYCN*$^{49806056..49807510}$,<br>*NCCRP1*$^{49800973..49804830}$
18	11 898 552	snp41877-scaffold546-944746	***KCNG4***$^{\mathbf{11894881..11909467}}$,<br>*NECAB2*$^{11690153..11731540}$,<br>*TAF1C*$^{11868350..11877938}$,<br>*HSDL1*$^{11832225..11846737}$,<br>*MBTPS1*$^{11784809..11829970}$,<br>*DNAAF1*$^{11846843..11868072}$,<br>*ADAD2*$^{11879617..11886040}$,

Chr	Position *	SNP	Genes **
			ATP2C2$^{12000229..12079250}$,<br>*WFDC1*$^{11951833..11978108}$,<br>*MEAK7*$^{12079026..12104623}$
18	3 837 951	snp6168-scaffold1217-1985930	***BCAR1***$^{3809751..3843150}$,<br>*LDHD*$^{3694393..3698425}$,<br>*BCNT*$^{3858817..3957257}$,<br>*P97BCNT*$^{3965191..3984379}$
20	66 737 512	snp16745-scaffold1753-34329	*SRD5A1*$^{66521371..66557879}$,<br>*NSUN2*$^{66558291..66590095}$,<br>*UBE2QL1*$^{66652430..66709023}$,<br>*MED10*$^{66776806..66785320}$
20	9 934 310	snp17084-scaffold1785-86716	***MCCC2***$^{9930596..10011105}$,<br>*CARTPT*$^{9879360..9881688}$,<br>*BDP1*$^{10017212..10101724}$
20	36 698 503	snp52283-scaffold776-291202	***WDR70***$^{36678460..36951048}$,<br>*GDNF*$^{36601218..36629923}$
20	7 640 897	snp57358-scaffold913-963263	*ARHGEF28*$^{7708446..8053019}$
21	15 802 894	snp23789-scaffold240-161570	*AKAP13*$^{15509421..15831062}$,<br>*KLHL25*$^{15858146..15908551}$
22	27 023 830	snp8-scaffold1-286588	*CNTN3*$^{27061216..27354315}$
24	33 188 152	snp1555-scaffold1042-1068020	*LAMA3*$^{33026004..33281542}$,<br>*ANKRD29*$^{33302633..33355333}$,<br>*NPC1*$^{33374981..33418847}$
24	41 365 838	snp54586-scaffold833-2411812	*PTPRM*$^{40689884..41344869}$,<br>*RAB12*$^{41427552..41449003}$,<br>*MTCL1*$^{41484249..41596722}$
26	2 502 077	snp34795-scaffold413-993146	*GLRX3*$^{2612246..2644263}$
26	29 391 796	snp41114-scaffold532-1082302	***BTRC***$^{29360210..29542837}$,<br>*FBXW4*$^{29220774..29308278}$,<br>*POLL*$^{29331268..29339687}$,<br>*DPCD*$^{29308767..29331223}$

Chr	Position *	SNP	Genes **
26	15 544 992	snp47523-scaffold67-1110214	*GFRA1*$^{15295458..15530217}$, *ATRNL1*$^{15632606..16441513}$
26	15 957 679	snp47533-scaffold67-1525470	***ATRNL1***$^{\mathbf{15632606..16441513}}$
26	16 947 347	snp47556-scaffold67-2509157	***ABLIM1***$^{\mathbf{16745034..17079215}}$, *AFAP1L2*$^{17113418..17229239}$
26	26 021 230	snp55577-scaffold861-1367391	***SORCS3***$^{\mathbf{25891651..26541837}}$
27	26 549 904	snp26261-scaffold275-1733756	*FRG1*$^{26375919..26390204}$, *ASAH1*$^{26324136..26353014}$
29	22 176 436	snp14507-scaffold1585-179608	*ANO5*$^{21904135..21999796}$
29	16 138 152	snp19092-scaffold192-2366	*TENM4*$^{16203644..16885831}$
29	16 134 831	snp32742-scaffold378-185653	*TENM4*$^{16203644..16885831}$
29	17 317 479	snp54961-scaffold840-1219604	***USP35***$^{\mathbf{17260319..17326282}}$, *GAB2*$^{17081591..17259528}$, *KCTD21*$^{17326470..17341773}$, *ALG8*$^{17388598..17414771}$, *KCTD14*$^{17458319..17464560}$, *INTS4*$^{17478869..17592447}$, *NDUFC2*$^{17421065..17429291}$, *THRSP*$^{17430132..17435304}$

* Position of SNP is indicated according to ARS1.2 genome assembly; ** Bold type indicates genes within which the identified SNPs are localized

Table S4 Functional annotation of candidate genes

Chr	Candidate genes	Gene function
1	*HACL1*$^{152427023..152470636}$	fatty acid oxidation, the process of fatty acid metabolism
	KNG1$^{80175447..80201003}$	blood clotting, negative regulation of blood clotting
	FETUB$^{80226559..80241470}$	sperm binding
	AHSG$^{80273479..80280715}$	ossification, negative regulation of bone mineralization, regulation of inflammatory response, regulation of phagocytosis
	HRG$^{80206861..80214843}$	positive regulation of the immune response
2	*NPPC*$^{16144029..16148670}$	body growth regulation
	PDE6D$^{16236483..16290580}$	visual perception, reaction to an irritant
	ADAM23$^{\mathbf{41016764..41207167}}$	development of brain tissue, parathyroid gland, enrichment of immune cells
3	*SLC27A3*$^{103529170..103534260}$	transport of fatty acids
	CREB3L4$^{103679930..103685385}$	spermatogenesis
	CRTC2$^{103662689..103672394}$	gluconeogenesis
	ROR1$^{39115242..39572757}$	sensory sound perception
4	*AOAH*$^{59458372..59656794}$	the process of fatty acid metabolism, the process of lipopolysaccharide catabolism,
5	***BICD1***$^{\mathbf{76054530..76298702}}$	response to the viral process
	AGAP2$^{55063063..55080580}$	development of breast alveoli
	MSRB3$^{47675346..47862677}$	It may affect the shape and size of the ears in pigs and sheep, the connection with growth in cattle
	HMGA2$^{47162168..47306445}$	the process of obesity induced by diet, the relationship with growth
	CRADD$^{\mathbf{23232979..23425649}}$	the "high-growing region" on chromosome 10 in mice is responsible for the growth and deposition of fat in pigs
9	*FIG4*$^{27692407..27859852}$	the behavior of the musculoskeletal system, the development of neurons
	HEY2$^{12796333..12808691}$	morphogenesis of the left and the right ventricle of the heart
	EYA4$^{58526657..58849760}$	sensory sound perception

Chr	Candidate genes	Gene function
	TCF21$^{59157639..59160660}$	lungs morphogenesis; pulmonary vessels, bronchioles and diaphragm development
	FYN$^{25894431..26111694}$,	forebrain development
	TRAF3IP2$^{26149367..26194655}$	heart development
10	*MAX* $^{25900233..25924465}$	positive transcription regulation from the promoter of RNA polymerase II
	GPX2$^{26053403..26056892}$	stress response
	TYRO3$^{66086217..66103879}$	development of sexual organs in males and females, innate immune response
	HOMER1$^{90737422..90876268}$	skeletal muscle contraction
11	***ANTXR1***$^{67083768..67344176}$	blood vessels development
	APOB$^{77590208..77630122}$	intrauterine embryonic development, spermatogenesis, nervous system development, cholesterol metabolism, fertilization, postembryonic development
13	*TMC2*$^{51886958..51986741}$	mechanical stimulus detection is involved in the sensory perception of sound
	APMAP$^{41943197..41969801}$	процесс биосинтеза
	ANGPT4$^{59493311..59543214}$	angiogenesis
15	*TPH1*$^{47860142..47897105}$	the metabolic process of the amino acid family, the process of serotonin biosynthesis, positive regulation of fat cell differentiation, bone remodeling, development of breast alveoli, regulation of hemostasis
16	***XPR1***$^{60399354..60607780}$	virus response
	PLOD1$^{40203194..40232944}$	epidermis development
17	***GOLGA3***$^{26739013..26785475}$	spermatogenesis
18	*P2RX2*$^{26626197..26629842}$	sensory sound perception
	LRCOL1$^{26602142..26609535}$	digestion, lipid catabolism
	MBTPS1$^{11784809..11829970}$	lipid metabolism process
	DNAAF1$^{11846843..11868072}$	lung development, determination of left-right asymmetry of the pancreas, digestive tract and liver
	ATP2C2$^{12000229..12079250}$	breast epithelium development
20	*NSUN2*$^{66558291..66590095}$	spermatids development

Chr	Candidate genes	Gene function
20	*CARTPT*[9879360..9881688]	feeding behavior of adult animals
26	*FBXW4*[29220774..29308278]	cartilage development
	DPCD[29308767..29331223]	spermatogenesis
	GFRA1[15295458..15530217]	nervous system development
	AFAP1L2[17113418..17229239]	response to the inflammatory process of tissues
	***SORCS3*[25891651..26541837]**	training, memory
27	*FRG1*[26375919..26390204]	organ muscles development
	ASAH1[26324136..26353014]	fatty acid metabolism
29	*TENM4*[16203644..16885831]	neurons development

The genes localized within the SNP are highlighted in bold

Table S5. SNP candidates selected on the results of GWAS common for two or more signs of growth and development at the age of 4 and 8 months

№	Chr	SNP position	Eight months		Four months	
			Trait	P	Trait	P
1	3	snp24555-scaffold2498-324452	**WH8m**	**2,93E-07**	WH4m	1,08E-05
		15 657 299	**RH8m**	**2,36E-06**	RH4m	4,12E-05
			**BL8m**	**4,80E-06**		
			**CW8m**	**9,93E-06**		
					CD4m	4,79E-05
					BW4m	5,41E-05
2	5	snp14251-scaffold157-188734	BW8m	1,26E-05	**BW4m**	**4,83E-07**
		19 499 085	BL8m	4,88E-05	BL4m	2,17E-05
			CP8m	2,80E-05		
					WH4m	4,23E-05
					RH4m	1,91E-05
3	5	snp37630-scaffold463-64670	BW8m	8,55E-05	**BW4m**	**3,72E-06**
		46 630 429				
4	5	snp38426-scaffold486-2412676	**BW8m**	**4,83E-06**	**BW4m**	**1,50E-06**
		22 879 856				
5	6	snp40083-scaffold511-2344051	**BW8m**	**3,01E-06**	**BW4m**	**8,73E-07**
		60 553 915			RH4m	3,17E-05
					WH4m	3,21E-05
					BL4m	7,10E-05
6	10	snp1448-scaffold104-1147808	**BW8m**	**4,56E-06**	BW4m	5,21E-05
		73 347 268				
7	10	snp49271-scaffold703-2391474	WH8m	2,01E-05	WH4m	7,59E-05
		10 276 674	BL8m	4,05E-05	BL4m	2,35E-05
			RH8m	7,83E-05	RH4m	2,30E-05
					**BW4m**	**4,14E-06**
8	13	snp31438-scaffold348-1638233	**RH8m**	**1,60E-07**	RH4m	2,52E-05
		76 566 792	**WH8m**	**7,62E-07**	WH4m	1,59E-05
			**BL8m**	**2,13E-06**	BL4m	2,02E-05
			CP8m	2,84E-05		
					RW4m	3,96E-05

					BW4m	5,80E-05
					CD4m	6,29E-05
9	16	snp8624-scaffold131-2001386<br>56 416 291	**BW8m**	**8,21E-06**	**BW4m**	**5,51E-07**
10	18	snp41877-scaffold546-944746<br>9 951 450	**CD8m**	**1,45E-06**	**CD4m**	**4,06E-06**
			**BL8m**	**1,56E-06**	BL4m	8,18E-05
			**WH8m**	**1,57E-06**		
			**RH8m**	**2,71E-06**		
			**CP8m**	**5,63E-06**		

SNPs selected for the design of molecular genetic test systems are shown in gray fill; signs for which a genome-wide confidence level is established according to the results of GWAS are shown in bold: BW (body weight), WH (withers height), RH (rump height), BL (body length), CP (chest perimeter), CW (chest width), CD (chest depth), RW (rump width)

Table S6 Candidate SNPs associated with the body weight at 4 and 8 months of age in Karachai goats

SNP	SNP	Address A_ID	AlleleA_ProbeSeq	Chr	MapInfo	Source Strand	SourceSeq
snp14251 scaffold 157-188734	[T/C]	60796360	CCAAAACACCAAGTCTGCTGGCTCCAGGTAATCTGAAGACTCAATATGCT	5	19 934 148 (Manifest) / 19 499 085 (GWAS)	TOP	CCCACAGAGGGTGGGAGAGACAGGAGGTGGGCGTTGCGGTTAGGAGGGCACATTAGCAGC[A/G]AGCATATTGAGTCT
snp38426 scaffold 486-2412676	[A/G]	15705507	AGCTGTTTTAAATCAGATTGTGTCTTTCAGCTTAAGCTATGTTCTGAGAC	5	23 345 368 (Manifest) / 22 879 856 (GWAS)	BOT	CCCTGCAATTACACACTATGGATTTTCAATAACACCTATCAGATTTTTTGTCCATTTATG[T/C]GTCTCAGAACATAGCTTAAGCTGAAAGACACAATCTGATTTAAAACAGCTAGGGGCTGAA
snp37630 scaffold 463-64670	[T/C]	25802440	CCTTGTTTAACGGATAGAGTAAGTCACATTTCCTGTTTTCCTCTTAGTCA	5	47 480 416 (Manifest) / 46 630 429 (GWAS)	TOP	TTAGCATTTGTCTTGAGGGTTTGAGGCCTTAGAAAATATTGTTTTTATCCTACAGAGTAC[A/G]TGACTAAGAGGAAAACAGGAAATGTGACTTACTCTATCCGTTAAACAAGGAATTTTTTTT
snp40083 scaffold 511-2344051	[A/G]	18716406	ATCTGTTCAAACTTTGTTCATGACATACAAAAGGACTGGGAGTGGGAGGT	6	62 949 329 (Manifest) / 60 553 915 (GWAS)	BOT	CTAATACCTATTTTGAGACTGGAGCTAAACTGAATACAGAGGATGACCTAAATCAGAATG[T/C]ACCTCCCACTCCCAGTCCTTTTGTATGTCATGAACAAAGTTTGAACAGATAAAAAACAAA

snp1448 scaffold 104-1147808	[T/C]	43609359	CTAGATGTCAGGTG TTGGGACAGGGGT GTAGAAGGGAGAT TTGAGAGCCA	10	25 854 668 (Manifest) / 73 347 268 (GWAS)	TOP	GCTTGAGCTACCCAGGTGT GATCCTCGCTCCACGGCAT GAGCTCAGAGGTGCTGCA AACC[A/G]TGGCTCTCAAA TCTCCCTTCTACACCCCTGT CCCAACACCTGACATCTAG ACCAAGAAGG
snp8624 scaffold 131-2001386	[A/C]	52698321	GTGATCCTTCGGAG GTTGTTCTTAAAAT TCACATTTCCACTC GAAGTTAT	16	57 408 452 (Manifest) / 56 416 291 (GWAS)	BOT	GAACTAACAGTACGTCTTT TAACATACTTGTACATGTG TAAGTCAGAACCATTAAA ATGC[T/G]ATAACTTCGAG TGGAAATGTGAATTTTAAG AACAACCTCCGAAGGATC ACGGGAAGATGG

Table S7. SNP candidates associated with the live weight of Karachai goats were used to construct a molecular genetic test system

SNP	Polymorphism	Chr	Name Description		Localization in the reference sequence NCBI: NC_030812.1
			**Gene**	**Protein**	
snp38426-scaffold486-2412676	A23345368G, rs268270492 G/A	5	*CRADD* - CASP2 and RIPK1 domain containing adaptor with death domain	death domain-containing protein CRADD	23,232,979 ... 23,425,649
snp37630-scaffold463-64670	T47480416C, rs268269710 A/G	5	*HMGA2* - high-mobility group AT-hook 2	high mobility group protein HMGI-C	47,162,168 ... 47,306,445
			MSRB3 - methionine sulfoxide reductase B3	methionine sulfoxide reductase B3	47,675,346 … 47,862,677
snp1448-scaffold104-1147808	A25854668G, rs268234545 A/G	10	*FUT8* - fucosyltransferase 8	fucosyltransferase 8	25,310,706 ... 25,629,694
			MAX - MYC associated factor X	MAX protein	25,900,233 ... 25,924,465
			RAB15 – Ras-Related Protein Rab-15	Ras-Related Protein Rab-15	26,026,720 ... 26,050,417

Table S8 SNP candidates analysis for the molecular genetic test systems design

Chromosome: 5<br>Reference sequence NCBI: NC_030812.1<br>Polymorphism: A23345368G (ARS1.2), rs268270492 G/A	
23344921	TCCCACTCAACTACTTGGCTTCTCTCTTTCTCTATCAAAGAGAGTGATCTTTAGACTGGAATGTGTAACATAAACATGGTTAAGAATAAA
23345011	TTGAAGCACAGAGGATCAGTGATGATAATAATAGCTGCAACTATAATTTTTTGAGATCTACTCAGGACCAAGTATTAGGAAAGATAATTT
23345101	AGTTCTGGTTTTTTTTTTTTTTCCCCAAATTCTTATGGCAGCTTTTGCATAGTAGAGATTATTGTCACCGAACCCATTTTACAGGTGAGA
23345191	ACACCAAGGCTCAGGAAGACTTAGTAACAGGCCTGAAGTTATACATCCAGTAAGTGATGGGCTGAGATTCAGATCCAGGCCTGACTGTTT
23345281	TGCCTGGACACCCTGCTGCCCATGCCTTTCAGCCCCTAGCTGTTTTAAATCAGATTGTGTCTTTCAGCTTAAGCTATGTTCTGAGAC**G**CA
23345371	TAAATGGACAAAAAATCTGATAGGTGTTATTGAAAATCCATAGTGTGTAATTGCAGGGGAAACAAAAATGGAATATCAAAATAATGAAAA
23345461	ACAAAGCCTGTTTTAATTTCCTTTTTAAAGGTTCAGTTTAGACTACCTCAGGCAGTCAGACGGTGATCCTCAGGACACTTGAGATTTTGT
23345551	TCTGCAAGTAAATGGGTGTAAATGAAAGAAAGGATCCTTCGGGCAGCAAAGGTTCACAAAGCATCACAAATTAGACACCCCCAACTCCCT
23345641	CCCTTCTTAATTATATAACTGGTAACCCTATCTTTTGACAGAGAATCCTATATGGCTCATAAACAAGTTAAATTAATCTAGGCTCAATTG
23345731	AGTAGATTCATATTTCTTTGAACTCCTTTACACAAATTATCGACTTTAGACAAATAGGCTTTGTTGTTGCTATTCAGCCGCCAAGTCCTG
Chromosome: 5<br>Reference sequence NCBI: NC_030812.1<br>Polymorphism: T47480416C (ARS1.2), rs268269710 A/G	
47479971	CCGGAAGAGCACCTGTGTGACCCAGGAGGCCTGTCTCCAGGTGCTCAACCACAGGTGGACTCCTCCCGCCCACAGGGGGCCCTGCCACTC
47480061	TGGTACTCCTCCTGGGAGTGGAGACATGGAGACCACTACAAGGTGCTACTGAGCCCTTTGAAAAGTGTGCAGTGTAGTGAAGAGTCAGGT
47480151	GGGGCTTGGACTGGAATCCTGCTTGCCCTATGAACTACCTTATAACACCATACTGAACTTCAGTTTCTCCATCTGTCAAATGGAAATTCT
47480241	CTCATGTTAGGATAGACAAATGAATTAATGCATATAAAGTGCTTACTTAGCCTATAGCAAGCACTCAATGAAAATTTCGGCTAGTATGAT
47480331	AACATTAACGTAATTTTCATTGTATTTAGCATTTGTCTTGAGGGTTTGAGGCCTTAGAAAATATTGTTTTTATCCTACAGAGTAC**A**TGAC
47480421	TAAGAGGAAAACAGGAAATATGACTTACTCTATCCGTTAAACAAGGATTTTTTTTTAAAGTGCTGACTTCATTGCTAAGTACTGTATCAG
47480511	ATCCCTCCCTTCCTTCATTCTTCTACATTTACTGTGTGTCTTTGAGGTGCTAGGCATTGCATTAGATTCTAGTAAAATAGCAATGAGACG
47480601	GGCAGATAGATTTCCTTCTGTCAGAGAATTTATGATTCATGGGGGAAAGACACATATTACACAGTACACCAAGGTATAGAACCAGGTGTA
47480691	AGTTATAGGCTCTACTGTAGTGTTCATGGAATATGGGGGATCTAATAGGGTTTGCAAGATCAACAAGACCATCCTAAATTTCCATTTTTG
47480781	AGACCAGTTCTCAAAAGAGTCGCAAAGAGTCACACACGACAGAGCAACTGAACAAAAACAACTCGAAAGAGTAAGTGACTTGATTTCCAA
Chromosome: 10<br>Reference sequence NCBI: NC_030817.1<br>Polymorphism: A25854668G (ARS1.2), rs268234545 A/G	
25854221	GCTCCACTGTGACAGCAGCAGACGATTTGATTTCCCTATTTCTTGTTTCTTTTTCTTATACCAAATTTTCTCTGCCTCCCTCCAAGATGG
25854311	GATCTGCAAGGCCTGGGGACCTTATTAATTCACAAGGAAGGCCAACAGAAGCAGGAAGAGCACTGACCAGCATGGCCCAGTGGCCCCATC
25854401	CTGTTCCTGACTGTACCTTGGGCTATAAGCCTTCCTGCTCCTCCACAGAGTGTGCATGGAAGGTGTGGAGAGGCCACGACACAGTCCACA
25854491	TCTCAGGACATAGCCAATGGTGTCAGGAAATGGACTAAGTGTAGCCAGCTTGAGGCCAACATTTTAGCTCAGTTTTCACAAGGTCACCTA
25854581	ATCTTTAAATTGGAGAGGATGGCAGGTGCTTGAGCTACCCAGGTGTGATCCTCGCTCCACGGCATGAGCTCAGAGGTGCTGCAAACC**A**TG
25854671	GCTCTCAAATCTCCCTTCTACACCCCTGTCCCAACACCTGACATCTAGACCAAGAAGGTCATTCCTTGAGTCAAACAGGTATTATTGCAA
25854761	GTGCCAACTCCAAGTTGTTGACGTTAGATTTTGTTTCAGAAAGATTCTCAGGCCTTTTCTGGTCTCAGGGGGATATTTGCAATCAATTAG
25854851	CAGTGTCTGCAATGGACACTGGAAAAGAAGTGCCAATGGTTTTGTACAGAATTACTGTCCTTAAAAAACACTTTCTTTTTATATTTACAG
25854941	TGCAGCAGTGCAACTATACGGCCAGTAGAGAACGCATGAACTTGTAATGACCAATAACCATATCAGGGCCTTCAGAGCATAGGAGGCAGA
25855031	ACAGGGACTTTCTTTATTACTGTTATTATCATTCAAACTTTTATTTTACGTTGGAATATAGCTGATTAACAACGTTGTGATAGTTTCAGG

Table S9 Modeling the definition of polymorphism in position: a) rs268270492; b) rs268234545; c)rs268269710

NC_030812.1	
23345518	TGACTGCCTGAGGTAGTCTAAACTGAACCTTTAAAAAGGAAATTAAAACAGGCTTTGT
23345460	TTTTCATTATTTTGATATTCCATTTTTGTTTCCCCTGCAATTACACACTATGGATTTT
	rs268270492-TM-A-Cy5
	Cy5-TATG**T**GTCTCAGAACATAGCTTAA-BHQ-2
	rs268270492-TM-G-TAMRA
	TAMRA-TATG**C**GTCTCAGAACATAGCTTAA-BHQ-2
	rs268270492-TM-F->
	ATAACACCTATCAGATTTTTTGTCCATT
23345402	CA**ATAACACCTATCAGATTTTTTGTCCATT**TATG**C (T)** GTCTCAGAACATAGCTTAAG
23345347	**CTGAAAGACACAATCTGATTTAAAACA**GCTAGGGGCTGAAAGGCATGGGCAGCAGGGT
	<-rs268270492-TM-R
	TGTTTTAAATCAGATTGTGTCTTTCAG
23345289	GTCCAGGCAAAACAGTCAGGCCTGGATCTGAATCTCAGCCCATCACTTACTGGATGTA
23345231	TAACTTCAGGCCTGTTACTAAGTCTTCCTGAGCCTTGGTGTTCTCACCTGTAAAATGG

a)

NC_030817.1	
25854833	TCCCCCTGAGACCAGAAAAGGCCTGAGAATCTTTCTGAAACAAAATCTAACGTCAACAACTTGG
25854769	AGTTGGCACTTGCAATAATACCTGTTTGACTCAAGGAATGACCTTCTTGGTCTAGATGTCAGGT
	rs268234545-TM-G-R6G
	R6G-CCA**C**GGTTTGCAGCACCTC-BHQ-1
	rs268234545-TM-A-FAM
	FAM-CCA**T**GGTTTGCAGCACCTC-BHQ-1
	rs268234545-TM-F->
	GGTGTAGAAGGGAGATTTGAGAG
25854705	GTTGGGACAGG**GGTGTAGAAGGGAGATTTGAGAG**CCA**T (C)** GGTTTGCAGCACCTCTGAGCTCA
25854644	TGCCG**TGGAGCGAGGATCACACCTG**GGTAGCTCAAGCACCTGCCATCCTCTCCAATTTAAAGAT
	<-rs268234545-TM-R
	CAGGTGTGATCCTCGCTCCA

25854580	TAGGTGACCTTGTGAAAACTGAGCTAAAATGTTGGCCTCAAGCTGGCTACACTTAGTCCATTTC
25854516	CTGACACCATTGGCTATGTCCTGAGATGTGGACTGTGTCGTGGCCTCTCCACACCTTCCATGCA

b)

NC_030812.1	
47480580	AGAATCTAATGCAATGCCTAGCACCTCAAAGACACACAGTAAATGTAGAAGAATGAAGGAAGGG
47480516	AGGGATCTGATACAGTACTTAGCAATGAAGTCAGCACTTTAAAAAAAAATCCTTGTTTAACGGA
	rs268269710-TM-A-FAM
	FAM-TCA**T**GTACTCTGTAGGATAAAAAC-BHQ-1
	rs268269710-TM-G-R6G
	R6G-TCA**C**GTACTCTGTAGGATAAAAAC-BHQ-1
	rs268269710-TM-F->
	GTCATATTTCCTGTTTTCCTCTTAG ⇩
47480452	TAGAGTAA**GTCATATTTCCTGTTTTCCTCTTAG**TCA**T(C)**GTACTCTGTAGGATAAAAACAATA
47480391	TTT**TCTAAGGCCTCAAACCCTCAAGA**CAAATGCTAAATACAATGAAAATTACGTTAATGTTATC
	<-rs268269710-TM-R
	TCTTGAGGGTTTGAGGCCTTAGA
47480327	ATACTAGCCGAAATTTTCATTGAGTGCTTGCTATAGGCTAAGTAAGCACTTTATATGCATTAAT
47480263	TCATTTGTCTATCCTAACATGAGAGAATTTCCATTTGACAGATGGAGAAACTGAAGTTCAGTAT

c)

Table S10. Primers and fluorescent probes developed for genotyping polymorphism in loci rs268270492, rs268234545, rs268269710

**Primers**	**Fluorescent probes**
rs268270492	
rs268270492-TM-F (ATA ACA CCT ATC AGA TTT TTT GTC CAT T)	rs268270492-TM-G-TAMRA (TAMRA-TAT GCG TCT CAGAACATAGCTTAA-BHQ-2)
rs268270492-TM-R (TGT TTT AAA TCA GAT TGT GTC TTT CAG)	rs268270492-TM-A-Cy5 (Cy5-TAT GTG TCT CAG AAC ATA GCT TAA-BHQ-2)
rs268234545	
rs268234545-TM-F (GGT GTA GAA GGG AGA TTT GAG AG)	rs268234545-TM-A-FAM (FAM-CCA TGG TTT GCA GCA CCT C-BHQ-1)
rs268234545-TM-R (CAG GTG TGA TCC TCG CTC CA)	rs268234545-TM-G-R6G (R6G-CCA CGG TTT GCA GCA CCT C-BHQ-1)
rs268269710	
rs268269710-TM-F (GTC ATA TTT CCT GTT TTC CTC TTA G)	rs268269710-TM-A-FAM (FAM-TC ATG TAC TCT GTA GGA TAA AAA C-BHQ-1)
rs268269710-TM-R (TCT TGA GGG TTT GAG GCC TTA GA)	rs268269710-TM-G-R6G (R6G-TC ACG TAC TCT GTA GGA TAA AAA C-BHQ-1)

Table S11. Indicators of meat productivity of young Karachai goats of different genotypes by SNP rs268269710, rs268270492, rs268234545 (8 months)

Indicator	Polymorphism/Genotype								
	rs268269710			rs268270492			rs268234545		
	TT	TC	CC	AA	AG	GG	AA	AG	GG
**Slaughter indicators, morphological composition of carcasses**									
Pre-slaughter weight, kg	32,81±0,59	34,16±0,37	35,73±0,42*	34,64±0,33	31,22±0,54	36,15±0,48**	34,92±0,37	34,83±0,21	33,82±0,46
Weight of the fresh carcass, kg	14,24±0,38	15,12±0,24	16,07±0,29	15,88±0,21	12,65±0,32	15,86±0,29	15,85±0,22	15,02±0,14	14,42±0,27
Internal fat weight, kg	0,96 ±0,06	1,12±0,12	1,20±0,17	1,00±0,04	0,89±0,06	1,17±0,09	1,15±0,10	1,10±0,04	1,00±0,07
Slaughter weight, kg	15,20±0,32	16,24±0,19	17,27±0,24*	16,88±0,12	13,54±0,24	17,03±0,12**	17,00±0,20	16,12±0,10	15,42±0,18
Slaughter yield, %	46,32±0,44	47,54±0,22	48,33±0,32*	48,73±0,28	43,37±0,32	47,11±0,44*	48,86±0,25	46,28±0,12	45,59±0,21
Boneless meat weight, kg	10,38±0,13	11,28±0,06	12,46±0,10**	12,22±0,05	10,32±0,10	12,21±0,08**	12,04±0,06	11,96±0,03	11,42±0,09
Bone and tendon weight, kg	3,32±0,16	3,64±0,07	3,70±0,09	3,61±0,08	3,35±0,05	3,59±0,04	3,45±0,06	3,49±0,04	3,37±0,04
The ratio of boneless meat to bones and tendons	3,13±0,12	3,10±0,07	3,37±0,10	3,38±0,06	3,08±0,09	3,40±0,11	3,49±0,11	3,42±0,08	3,39±0,09
Loin eye area, cm^2	12,44±0,17	12,46±0,09	13,09±0,14*	13,01±0,04	11,47±0,10	13,21±0,12**	12,26±0,12	12,08±0,06	11,95±0,10
**The content of minced meat in an average sample, %**									
Moisture	69,40±0,68	68,55±0,72	67,20±0,67	69,14±0,59	69,21±0,72	66,90±0,41	68,72±0,68	68,25±0,72	69,05±0,67
Fat	8,75±0,21	9,23±0,26	9,12±0,18	8,65±0,19	8,41±0,26	9,62±0,18	9,10±0,17	8,88±0,26	8,06±0,18
Ash	1,0±0,04	1,0±0,05	1,1±0,07	1,0±0,05	1,0±0,05	1,1±0,06	1,0±0,05	1,0±0,06	1,0±0,05
Protein	20,90±0,21	21,20±0,34	22,56±0,17	21,21±0,27	22,34±0,43	22,48±0,52	21,16±0,23	21,87±0,43	21,87±0,27
Caloric content, kcal/100 g	167,06±0,39	172,94±0,47	177,38±0,55	167,40±0,40	170,01±0,34	181,63±0,63	171,47±0,39	172,24±0,47	164,71±0,32

Significance levels: * - $p < 0.05$; ** - $p < 0.01$
